# Mutagenic Effect of Three Ion Beams on Rice and Identification of Heritable Mutations by Whole Genome Sequencing

**DOI:** 10.3390/plants9050551

**Published:** 2020-04-26

**Authors:** Yunchao Zheng, Shan Li, Jianzhong Huang, Haowei Fu, Libin Zhou, Yoshiya Furusawa, Qingyao Shu

**Affiliations:** 1 National Key Laboratory of Rice Biology, Institute of Crop Sciences, Zhejiang University, Hangzhou 310058, China; yunchao.zheng@zju.edu.cn (Y.Z.); lishan@zju.edu.cn (S.L.); 2 Institute of Nuclear-Agricultural Sciences, Zhejiang University, Hangzhou 310058, China; jzhuang@zju.edu.cn; 3 Zhejiang Provincial Key Laboratory of Crop Germplasm, Zhejiang University, Hangzhou 310058, China; 4 Jiaxing Academy of Agricultural Science, Jiaxing, Zhejiang 314016, China; fhw670225@sina.com; 5 Biophysics Group, Biomedical Research Center, Institute of Modern Physics, Chinese Academy of Science, Lanzhou 730000, China; libinzhoulz@gmail.com; 6 Department of Basic Medical Sciences for Radiation Damages, National Institute of Radiological Sciences, National Institutes for Quantum and Radiological Science and Technology, Chiba 263-8555, Japan; furusawa448@mac.com

**Keywords:** rice (*Oryza sativa* L.), mutagenesis, mutation breeding, ion beams, whole genome sequencing, mutation spectrum

## Abstract

High-energy ion beams are known to be an effective and unique type of physical mutagen in plants. However, no study on the mutagenic effect of argon (Ar) ion beam radiation on rice has been reported. Genome-wide studies on induced mutations are important to comprehend their characteristics for establishing knowledge-based protocols for mutation induction and breeding, which are still very limited in rice. The present study aimed to investigate the mutagenic effect of three ion beams, i.e., Ar, carbon (C) and neon (Ne) on rice and identify and characterize heritable induced mutations by the whole genome sequencing of six M_4_ plants. Dose-dependent damage effects were observed on M_1_ plants, which were developed from ion beam irradiated dry seeds of two *indica* (LH15, T23) and two *japonica* (DS551, DS48) rice lines. High frequencies of chlorophyll-deficient seedlings and male-sterile plants were observed in all M_2_ populations (up to ~30% on M_1_ plant basis); plants from the seeds of different panicles of a common M_1_ plant appeared to have different mutations; the whole genome-sequencing demonstrated that there were 236–453 mutations in each of the six M_4_ plants, including single base substitutions (SBSs) and small insertion/deletions (InDels), with the number of SBSs ~ 4–8 times greater than that of InDels; SBS and InDel mutations were distributed across different genomic regions of all 12 chromosomes, however, only a small number of mutations (0–6) were present in exonic regions that might have an impact on gene function. In summary, the present study demonstrates that Ar, C and Ne ion beam radiation are all effective for mutation induction in rice and has revealed at the genome level the characteristics of the mutations induced by the three ion beams. The findings are of importance to the efficient use of ion beam radiation for the generation and utilization of mutants in rice.

## 1. Introduction

Heavy ion beams are a new type of physical mutagen that is different from the traditionally used γ radiation in many aspects [1]. They are featured with high LETs (linear energy transfer) and have been demonstrated to induce mutations at a higher frequency, with a wider spectrum and more efficiency [2]. In rice, Yamaguchi et al. [3] first performed a comparative study on the mutagenic effects of helium (He) and carbon (C) ion beams (with LETs of 76 keV/μm and 220 keV/μm, respectively) with γ rays. They demonstrated that the efficiency of ion beams either equaled or exceeded that of γ rays and that the mutation rate of ion beams was higher than that of γ rays. C ion beams have since been used in rice breeding programs and several valuable mutant lines have been developed, such as low cadmium rice [4], ultraviolet-B (UVB)-tolerant mutant [5] and extremely late heading mutants [6].

To comprehend the characteristics and frequencies of the mutations induced by mutagens, next generation sequencing (NGS) technologies have been deployed for sequencing the genomes of plants derived from mutagenic treatment. For the chemical mutagen ethyl methyl sulfone (EMS), a genome-wide analysis of induced mutations has been performed in rice [7] and a number of other plant species, e.g., wheat [8,9,10], foxtail millet [11], *Chenopodium quinoa* Willd. [12], sorghum [13], tomato [14], *Cucurbita pepo* [15], peach [16], *Lotus japonicus* [17] and *Jatropha curcas* [18]. Similar studies have also been performed for physical mutagens, such as γ ray mutagenesis in rice [19,20] and poplar [21], fast neutron mutagenesis in *Arabidopsis thaliana* [22] and rice [23], and ion beams in *Arabidopsis thaliana* [24,25,26,27,28]. In *Arabidopsis*, a comparative analysis showed that C ion radiation could induce more single base substitutions (SBSs) and short insertion/deletions (InDels) than argon (Ar) ion radiation, while the latter generated chromosomal rearrangements and/or large deletions more frequently than the former [28].

Recently, the mutations induced by ion beams have also been investigated in rice by whole genome or exome sequencing. Using an exome-sequencing procedure, Ichida et al. [29] analyzed the characteristics and distribution of the mutations induced by C ion beams in the absence of bias introduced by the visual mutant selections. They observed that the number of mutations within the target exon regions was 9.06 ± 0.37 (average ± SE) in unselected M_2_ lines, which were derived from dry seeds treated with 150 Gy C ion beam (^12^C^6+^, 135 MeV/u, LET: 23–30 keV/μm), and the mutation frequency increased with the increase of the irradiation dose. Two groups further independently performed comparative analyses of the mutations induced by C ion beams and γ rays by whole genome sequencing [30,31]. Unlike Ichida et al. [29], both studies analyzed the advanced mutant lines (M_4_–M_6_) of a *japonica* [30] and *indica* rice [31], respectively. The conclusions of the two studies were quite consistent: both mutagens induced SBSs and small InDels, and γ rays induced more variations (particularly SBSs), but less structural variations (SVs) on average than the C ion beams. However, Li et al. [30] observed no significant differences of the proportion of InDels (~30%) in plants developed from two mutagens, while Yang et al. [31] reported a higher proportion of InDels in C ion beam induced plants (25.44%) than in those from the γ rays treatment (17.85%). Besides, SVs were only identified in one study [30].

In addition to the commonly used C ion beams, there are other types of ion beams, i.e., nitrogen (N), Ar, He, neon (Ne) and iron (Fe) ion beams, that can be used for plant mutagenesis [1]. However, thus far in rice, only C and He ion beams have been used for mutation induction, though other types of low energy ion beams (known as ion implantation, e.g., N) have already widely been used [32]. In the present study, the mutagenic effect of three ion beams, i.e., argon (^40^Ar^18+^), carbon (^12^C^6+^) and neon (^20^Ne^10+^) were investigated using two *indica* and two *japonica* rice lines, and the genome-wide mutations were identified by whole genome sequencing of six advanced mutant lines and the characteristics were further analyzed using bioinformatic tools, with the aim to understand more about the characteristics and usefulness of ion beam mutagenesis in rice.

## 2. Results

### 2.1. Effect of Ion Beam Radiation on M_1_ Plants

**Figure 1:**
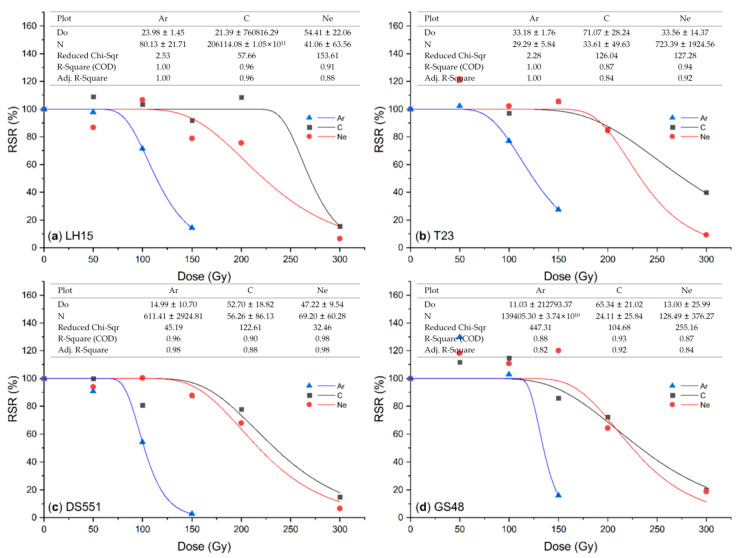
Relative seedling survival rate (RSR) of the M_1_ seedlings from the dry seeds of rice lines (**a**) LH15, (**b**) T23, (**c**) DS551, and (**d**) GS48 irradiated with the argon (Ar), carbon (C) and neon (Ne) ion beams. RSR is present as % of the seedling survival rate of each treatment divided by that of their respective untreated controls. Scatters are experimental data points and fitted by nonlinear curves based on a single-hit multi-target (SHMT) model [33]. The SHMT model equation is S = (1 − (1 − exp(−D/Do))^N^) × 100.

All three ion beams showed damage effects on M_1_ plants in a dose-dependent mode, with Ar being more effective than C and Ne ion beams. Radiation severely affected seedling growth and obviously reduced their survival rate at a higher dose of treatment (Figure 1), with significant effects of radiation type and dose as well as genotype. Among the three ion beams, Ar ion beams seemed much more damaging than the other two. The relative seedling survival rate (RSR) was reduced to ~20% (LH15, T23 and GS48) to almost zero (DS551) when irradiated with 150 Gy of Ar ion beams, while similar RSR reduction levels were observed for 300 Gy of C and Ne ion beams (Figure 1). Overall, C and Ne seemed to have more similar dose effects than Ar radiation on RSR and all dose effect curves had a “shoulder” before they sharply fell (Figure 1).

**Figure 2:**
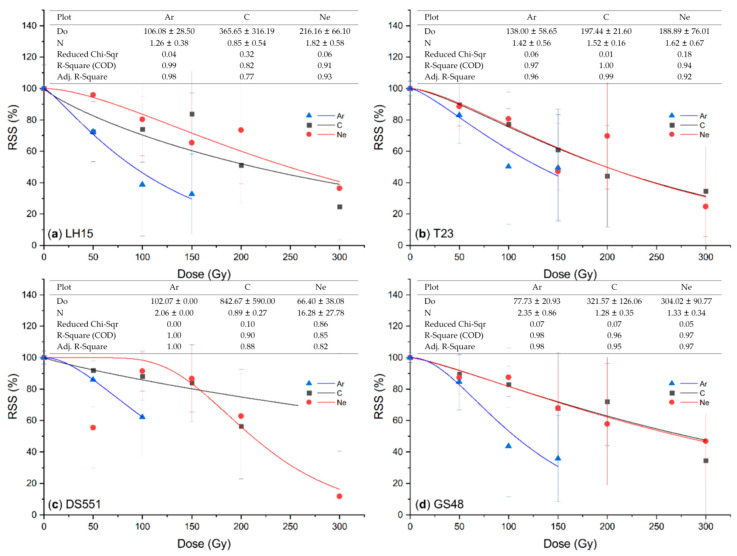
Relative seed set (RSS) of the M_1_ plants developed from the dry seeds of rice lines (**a**) LH15, (**b**) T23, (**c**) DS551, and (**d**) GS48 irradiated with the argon (Ar), carbon (C) and neon (Ne) ion beams. RSS is present as % of the seed set of each treatment divided by that of their respective untreated controls. Data are the mean ± standard deviation of the seed set of 10 plants. Scatters are experimental data points and fitted by nonlinear curves based on a single-hit multi-target (SHMT) model [33]. The SHMT model equation is S = (1 − (1 − exp(−D/Do))^N^) × 100.

Radiation also substantially reduced the seed set of M_1_ plants (Figure 2). Firstly, a great variation of the seed set was observed among the different panicles of a single M_1_ plant and among the different plants treated with the same ion beams at the same dose, which was manifested by the great standard variation of the relative seed set at each data point. Secondly, there was a tendency of decline for the seed set with the increase of radiation dose for all four genotypes. Ar again seemed to be the most effective one, with the reduction of the seed set at 150 Gy approaching to the same level of the other two at 300 Gy in most cases. Except for Ne ion radiation for DS551, the dose effect curves had no typical “shoulder”, and the curves of C and Ne ion radiation were similar to each other in all four genotypes except DS551 (Figure 2).

### 2.2. Morphological Mutants in M_2_ Populations

**Figure 3:**
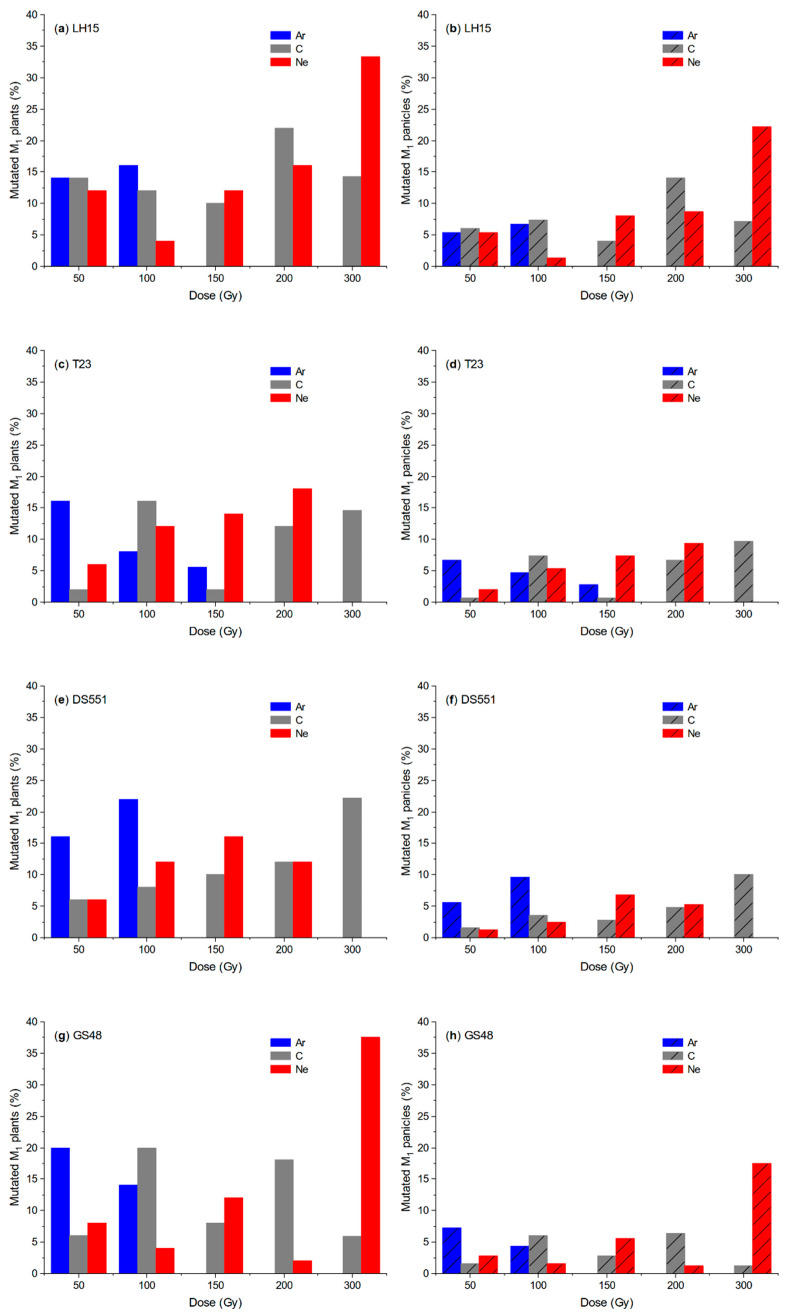
Frequency of chlorophyll (Chl)-deficient mutations in the M_2_ populations of four rice lines on the M_1_ (**a**,**c**,**e**,**g**) plant-row and (**b**,**d**,**f**,**h**) the panicle-row basis. M_1_ plants were grown from the dry seeds irradiated with the different doses of argon (Ar), carbon (C) and neon (Ne) ion beams. Chl-deficient plants were determined during the seedling stage.

**Figure 4:**
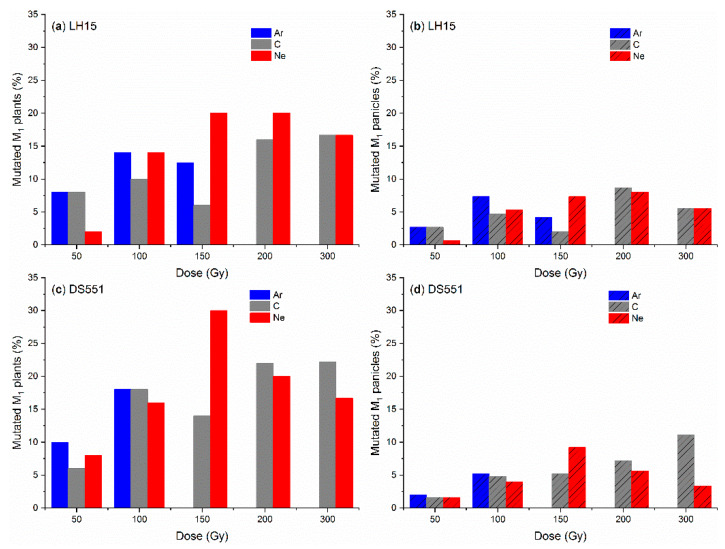
Frequency of male-sterile mutations in the M_2_ populations of the two rice lines on the M_1_ (**a**,**c**) plant-row and (**b**,**d**) the panicle-row basis. M_1_ plants were grown from the dry seeds irradiated with the different doses of argon (Ar), carbon (C) and neon (Ne) ion beams. Male-sterile plants were determined during the flowering stage.

Chlorophyll (Chl)-deficient mutants (mostly albino or yellow leaf mutants) and morphological mutants (e.g., male-sterile mutants) were observed in all the M_2_ populations (Figure 3 and Figure 4). However, there were only a limited number of the M_2_ plants at high doses (Ar: 150 Gy; C and Ne: 300 Gy) due to the low survival rate and low fertility of the M_1_ plants, hence the mutation frequencies at these doses could be over/under estimated. Similar to its effect on M_1_ plants, greater mutation frequencies were observed for Ar than for C and Ne radiation at the dose of 50 and 100 Gy in most cases. A few exceptions were Chl deficiency at 100 Gy for T23 (Figure 3c,d) and GS48 (Figure 3g,h), and Chl deficiency for LH15 on the M_1_ panicle basis (Figure 3b). At higher doses of radiation, mutation frequencies reached up to ~22–37% on the M_1_ plant basis and ~15–25% on the M_1_ panicle basis for both Chl deficiency and male sterility, but without consistent and significant ion beam/genotype/dose effect (Figure 3 and Figure 4), though there were two specific cases where the mutation frequency increased with the increase of radiation dose, i.e., the Chl deficiency of Ne irradiated T23 (Figure 3c,d) and C irradiated DS551 (Figure 3e).

**Figure 5:**
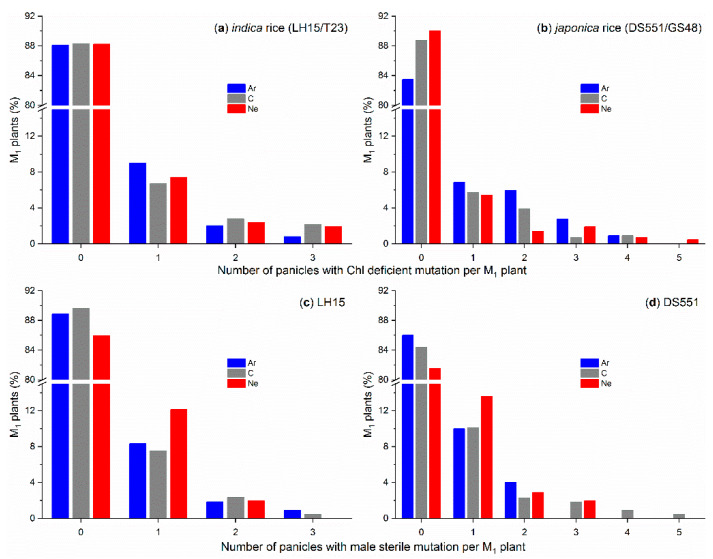
Frequency of the M_1_ plants of *indica* rice lines (LH15/T23) as well as the *japonica* rice lines (DS551/GS48) having different number of panicles with (**a**,**b**) chlorophyll (Chl)-deficient and (**c**,**d**) male-sterile mutation per M_1_ plant. M_1_ plants were grown from the dry seeds irradiated with the different doses of argon (Ar), carbon (C) and neon (Ne) ion beams. Chl-deficient and male-sterile plants were determined during the seedling and flowering stages, respectively. Three and five panicles were harvested from each M_1_ plant for the *indica* and *japonica* rice, respectively.

Three and five panicles were harvested from each M_1_ plant for *indica* and *japonica* rice, respectively. Not all the panicles of one M_1_ plant had mutated M_2_ plants, and in most cases, only one or two panicle rows of one M_1_ plant had mutated plants (Figure 5). Therefore, the mutation frequencies on the panicle basis were much lower than on the plant basis for all treatments (Figure 3 and Figure 4).

Plants with other mutated traits such as dwarfism were also observed in the M_2_ populations but with much lower frequencies than the male-sterile plants. High frequencies of dwarf mutants were observed in the M_2_ populations of the *japonica* line DS551, but almost no dwarf mutants were observed in the *indica* rice LH15. Both male-sterile and dwarf mutations were proven to be inheritable: progenies of dwarf plants showed the same trait in M_3_ and M_4_ progenies, while a proportion of the M_3_ progenies derived from the wild-type (WT) sibling plants of the M_2_ male-sterile plants were always male-sterile.

### 2.3. Genome-wide Mutations in M_4_ Plants

A total of 137–172 million clean reads were generated per sample and when mapped to the reference Nipponbare genome, it was equivalent to a mapping rate of 95.63–98.79% (Table 1). These reads covered 97.29–97.41% of the whole reference genome with a sequencing depth of 53.46–66.52× (Table 1). A total of 236–453 SBSs and InDels were identified in the six M_4_ mutant plants, with more SBSs than InDels in all the plants (Table 2)

**Figure 6:**
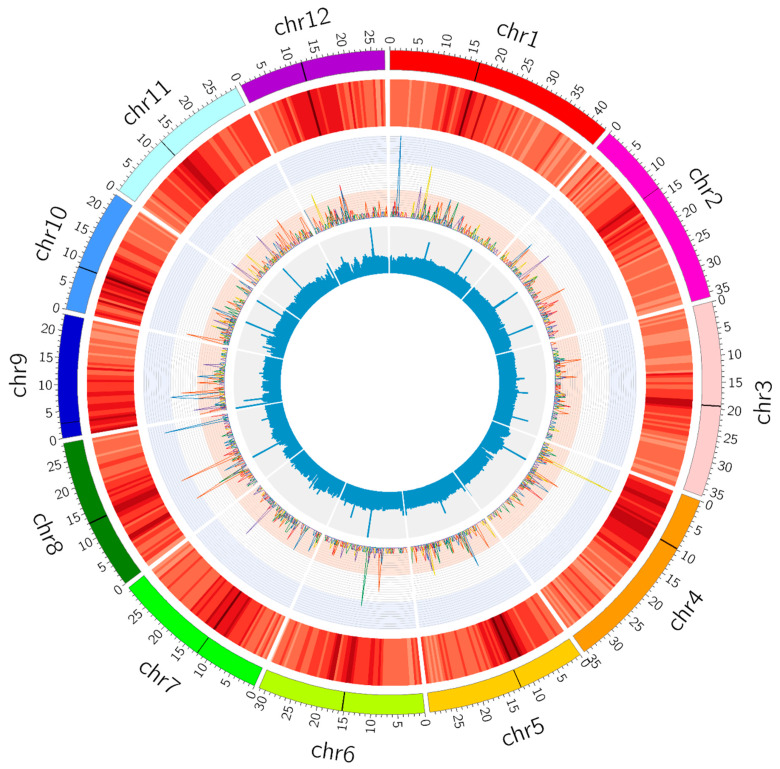
Genome-wide characterization of the single base substitutions (SBSs) and small insertion/deletions (InDels) in the six ion beam-mutagenized M_4_ rice plants. Circles from outside to inside: Twelve rice chromosomes on the 1-Mb scale with the black band standing for centromere; Heat map of the repetitive sequence contents in the rice reference genome in non-overlapping 500-kb windows, the darker the color, the higher the content, with repetitive sequence data derived from the Rice Genome Annotation Project (RGAP) release 7; Line plots of the SBS and InDel numbers in the non-overlapping 500-kb windows (the highest peak equates to 35 mutations in a 500-kb window on the chromosome 1 of the Ne_50 plant), the line colors are red for Ar_50, orange for Ar_100, yellow for C_150, green for C_200, blue for Ne_50, and purple for Ne_100, respectively; Blue histograms represent the average sequencing depth of all the rice samples in the non-overlapping 500-kb windows.

SBSs and InDels were distributed, not completely evenly, in all the chromosomes of the rice genome (Figure 6). There were a few peaks where more variations existed in 500-kb windows, e.g., the highest peak on the chromosome 1 of the Ne_50 plant with 35 mutations (Figure 6). Repetitive sequences were often present around centromeric regions, but these regions seemed not always to have more mutations, and indeed many peaks were in non-repetitive regions (Figure 6).

**Figure 7:**
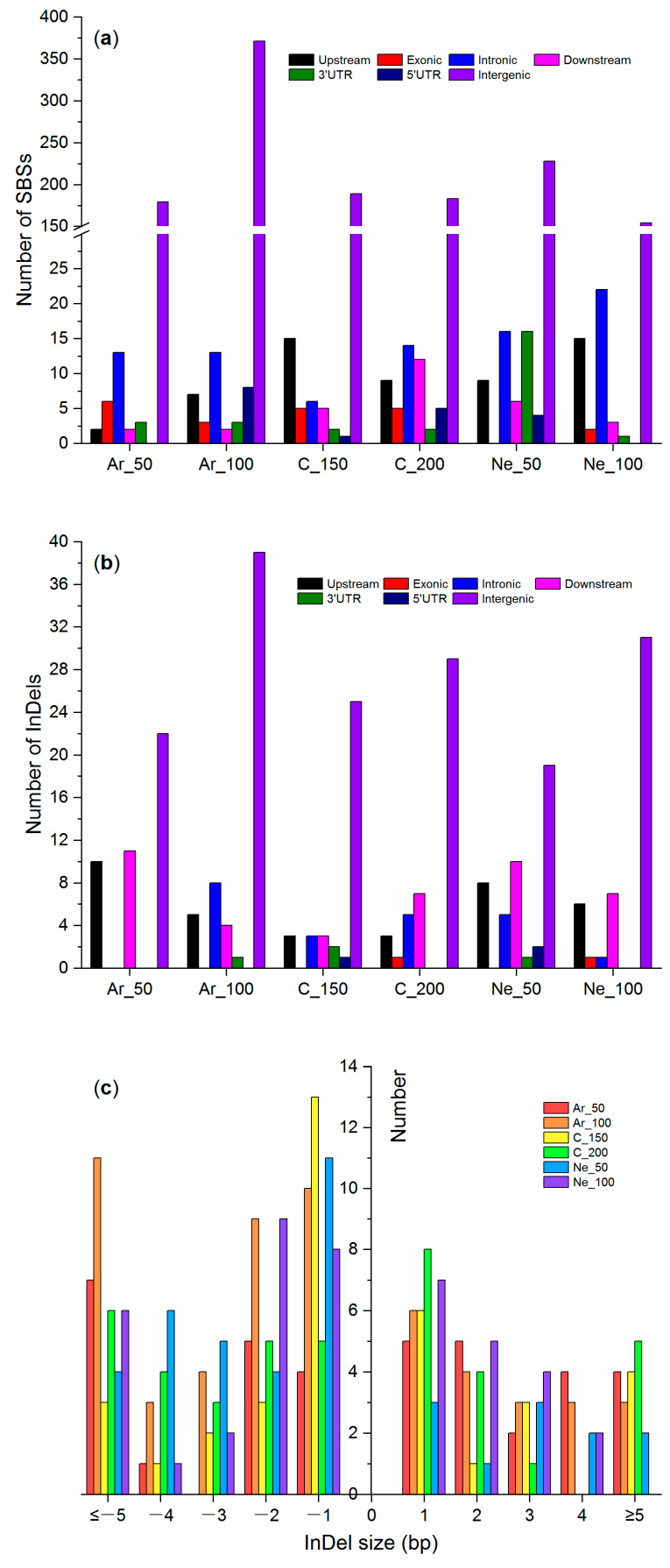
Numbers of (**a**) the single base substitutions (SBSs), (**b**) the insertion/deletions (InDels) located in the different genomic regions and (**c**) the number of InDels with different sizes in the six ion beam-mutagenized rice M_4_ plants.

Among the six mutant plants, Ar_100 seemed to have far more SBSs and more InDels than other plants (Table 2). All six possible types of substitution were detected, with the transitions (Ti: C:G>T:A and T:A > C:G) to transversions (Tv: C:G > A:T, C:G > G:C, T:A > G:C, and T:A > A:T) ratios ranging from 1.31 (Ne_50) to 2.78 (Ar_100) (Table 2). SBSs and InDels were identified in the different genomic regions across the whole genome, but most of them were in the intergenic regions (Figure 7a,b). The size of the InDels varied from 1 to 50 bps, but the majority of them were 1–4 bps and more deletions were observed than insertions in all the mutant plants (Figure 7c).

Only a fraction of SBSs (0–2.9%) and InDels (0–2.4%) were in exonic regions (Table 2). In total, only 13 SBSs and two InDels could potentially either change encoded amino acids (nonsynonymous mutations) or truncate encoded peptides (frame-shift mutations). The 13 SBSs and two InDels that could have a functional effect on the respective genes were all verified using the integrative genomics viewer (IGV) [34] (Figures S1–S15).

## 3. Discussion

High-energy ion beam radiation is a new type of physical mutagen but its application in plant breeding has not been as common as γ rays, probably due to the limited accessibility to many plant breeders, particularly outside of Japan and China. In rice, there have been several reports on C/Ne ion beam mutagenesis and on the genetic, genomic and phenotypical characteristics of induced mutants. The present study not only evaluated the mutagenic effect of a new ion beam (^40^Ar^18+^) in parallel with C and Ne beams on rice from breeding perspectives, but also identified and characterized the mutations in advanced lines using whole genome sequencing. The findings of the present study hence have important implications to rice ion beam radiation breeding.

### 3.1. Ar, C and Ne Ion Beams Could All Be Used for Mutation Induction and Breeding in Rice

Ion beam radiation differs from γ rays because of its far greater LETs. The greater the LET, the more localized and denser ionizations would be produced in a cell, which could cause more DNA damages [1]. Although the LET of the Ar ion beam is about three and seven times more than that of C and Ne beams (Table 3), they all seemed to be effective for mutation induction when a suitable dose was applied. The dose curves of the seedling survival rates of M_1_ plants showed that there were “shoulder” doses where radiation showed no or limited negative effects, e.g., 50–150 Gy for C and Ne ion beams (Figure 1). Furthermore, a big shoulder of fitting curves occurred under low LET (C, Ne ion beams), whereas the shoulder became small with the increase of LET (Ar ion beams) (Table 3; Figure 1). A similar phenomenon was also presented in the survival curves of multicellular spheroids fitted by a single-hit multi-target (SHMT) model [33]. However, no such “shoulder” dose existed for the seed set except for the Ne ion beam radiation of DS551 (Figure 2), suggesting that the biological basis of radiation on growth and reproduction might be different. Based on these dose effect curves, we recommend that the doses for Ar, C and Ne radiation to be 100, 200 and 200 Gy, respectively. When the dry seeds were treated with ion beams using the above recommended doses, 50% relative seedling survival rate and seed set were achieved for most rice genotypes (Figure 1 and Figure 2).

At the phenotypical level, relative high mutation rates were achieved for all radiation in all four genotypes. The Chl deficiency mutation rates were similar to those reported by Yamaguchi et al. [3] on the M_1_ plant basis, e.g., about 8–15% for the two C and one He ion beam radiation at doses that attained 50–90% seedling survival rates. Similarly, the high mutation frequencies of male-sterile plants were observed in the populations of two rice lines, further demonstrating the effectiveness of these three ion beams for the mutation induction in rice. No previous studies had data of morphological mutations at a mature stage, hence they could not be compared directly with other studies on ion beam radiation in rice.

At the genomic level, the Ar_100 plant had substantially more SBSs and InDels than the other plants treated with other ion beams or doses (Table 2). However, the mutational impacts seemed to be not that different from each other, because the numbers of exonic mutation in the six plants were indeed quite similar (Table 2).

### 3.2. How to Make Full Use of Irradiated Seeds?

Compared with γ rays, ion beam radiation is not only far more expensive, but also far less available and accessible to plant breeders. Every irradiated seed is very precious and thus it is of paramount importance for plant researchers to make full use of mutagenized seeds in their programs.

Panicles are differentiated from shoot apex meristem (SAM), however, it is not yet known whether there is a progenitor cell or cells already in a mature seed, which will be eventually developed into panicles. If all panicles of a plant were developed from a single common progenitor cell in a seed, then all the panicles would carry the same mutations induced by the ion beam radiation. If there was more than one progenitor cell, different panicles could have different mutations. In the present study, we observed that among the five or three panicle rows of a M_1_ plant, most plants had one or two panicle rows with mutated seedlings at the seedling stage or mutated plants at the later stages (Figure 5), suggesting that different panicles are highly likely to be from different progenitor cells in seeds.

Based on the proportion of M_1_ plants with different numbers of panicles with mutations (Figure 5) and the probability analysis of different scenarios with one to six progenitor cells, each producing two or three panicles, it was reasoned that there were three to four progenitor cells in the two *indica* lines (with fewer panicles per plant) and four to five in the two *japonica* lines. Therefore, to make full use of the ion beam irradiated seeds for mutation breeding, M_2_ seeds should be harvested from all the panicles of the M_1_ plants because they could all carry different induced mutations.

### 3.3. Mutations Identified via Genome/Exome Sequencing

During the past 10 years, several studies have investigated genome-wide mutations induced by ion beams in rice and other plant species. In rice, Ichida et al. [29] investigated genome-wide mutations induced by C and Ne ion beams using exome sequencing of randomly selected M_2_ plants, while Li et al. [30] and Yang et al. [31] sequenced whole genomes of stable mutant lines at the generation of M_4_–M_6_ for the comparison of the mutations induced by C ion beams and γ rays. The overall results of these studies were consistent with the present study in terms of the type of induced mutations and their genome-wide distribution. For the quantitative analysis of the number of mutations, however, the present study can only be compared with those of Li et al. [30] and Yang et al. [31]. Due to the differences of materials and method used, the present study cannot be compared with Ichida et al. [29].

Li et al. [30] sequenced seven M_5_ lines of a *japonica* rice Hitomebore irradiated with 30 Gy ^12^C^6+^ beams (LET: 107 keV/μm). On average, 43.7 SBSs, 13.6 deletions and 5.3 insertions were detected in each mutant. Yang et al. [31] sequenced six (four M_4_, two M_5_) mutants of an *indica* rice R173 treated with 80 Gy ^12^C^6+^ beams (LET: 50 keV/μm). On average, 30.3 SBSs (including multiple nucleotide variants) and 10 InDels were observed for each of the four M_4_ mutant plants. In the present study, about 3–4 times more SBSs and InDels were observed in the M_4_ plants irradiated with 150–200 Gy of C ion radiation (Table 2). This discrepancy could be partially explained by the higher radiation doses applied in the present study, and partially by other factors such as the different varieties used in different studies.

**Figure 8:**
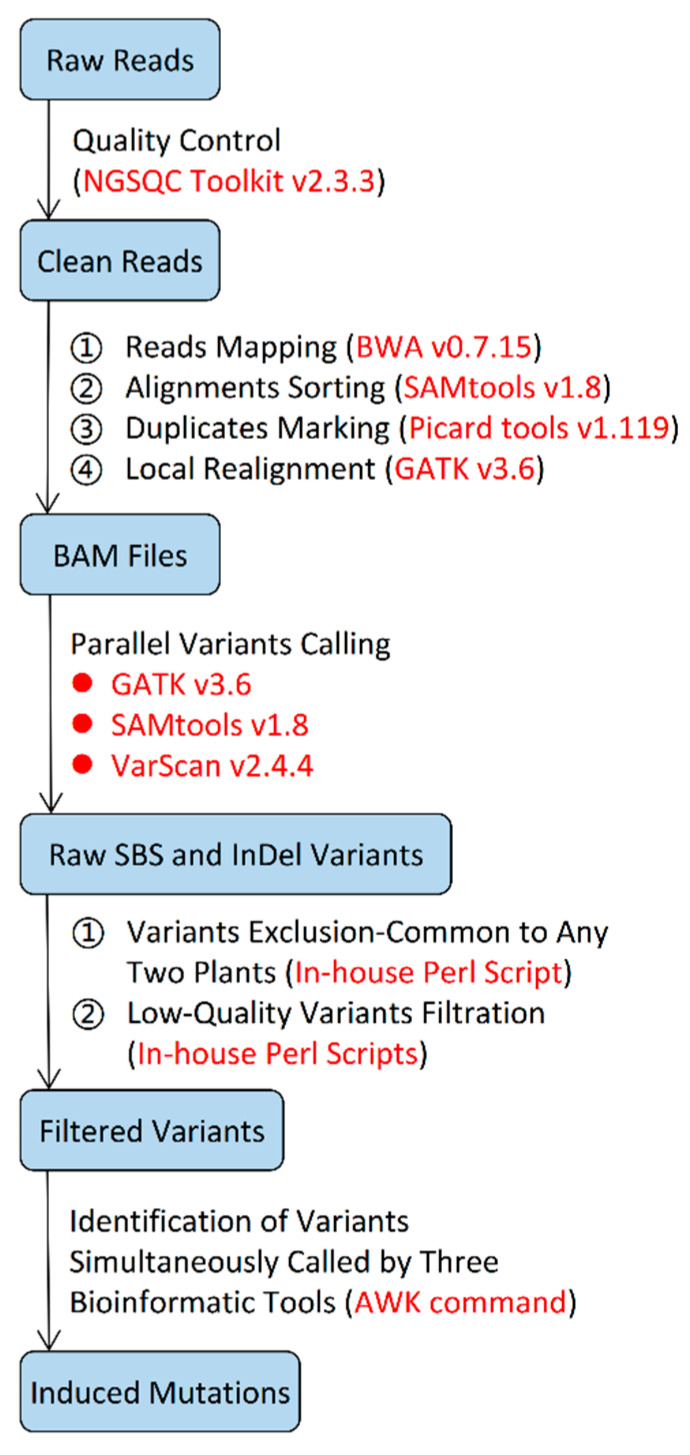
The pipeline used for the identification of the mutations induced by the three ion beams in the rice plants based on their whole genome-sequencing data. Blue box: Input/Output data; The actions taken and corresponding bioinformatic tools/scripts/Linux commands (highlighted by red color) used are presented for each step.

Methodologically, the present study not only excluded background mutations by removing mutations common to any two plants, but also minimized false positives by having the mutations identified by three different bioinformatic tools (Figure 8). As further confirmed by using the IGV program [34], the identified mutations were high likely to be true, though we did not verify them via wet experiment.

Due to its high LET nature, ion beam radiation is expected to generate SVs [1]. SVs were demonstrated in *Arabidopsis* in earlier studies [24,27]. Recently, SVs have also been identified in rice [29,30], though no SV was identified by Yang et al. [31] in their study. We also expect SVs to be present in the mutant plants developed in the present study and will perform detailed analysis separately.

## 4. Materials and Methods

### 4.1. Irradiation of Ion Beams and Mutant Development

The dried seeds of two *indica* (LH15 and T23) and two *japonica* (DS551 and GS48) rice lines were irradiated with Ar, C and Ne ion beams with 5 doses and different dose rates (Table 3) in the Heavy-Ion Medical Accelerator in Chiba (HIMAC) Facility at the National Institute of Radiological Science, Japan.

In October 2013, the irradiated seeds (M_1_) were sown together with the unirradiated controls. The survived M_1_ seedlings and their control ones were individually transplanted in a paddy field in the Winter Breeding Nursery of Zhejiang University in Lingshui, Hainan Province. At the seedling stage, the seedling survival rate was examined, and at mature stage, 10 M_1_ rice plants were randomly selected for the measurement of plant height and the number of tillers per plant. Seed set rates were assessed by the analysis of two panicles from each of the 10 M_1_ plants per treatment and respective controls. M_2_ seeds were harvested from individual M_1_ plants on a panicle (three panicles per plant for the two *indica* lines and five for the two *japonica* ones) and plant basis. Nonlinear dose–effect curves were drawn by fitting the experimental data with the SHMT model [33].

In 2014, M_2_ seeds were sown on a panicle and plant basis in the experimental paddy field of Jiaxing Academy of Agricultural Science, Zhejiang Province. Chl-deficient (albino or yellow) and male-sterile mutants are well-known to be the most common phenotypical mutations; hence they were investigated as representative traits for the assessment of the mutagenic effect. At the seedling stage, Chl-deficient mutants were observed on panicle basis at the seedling stage within two weeks after sowing. Thirty-six survived seedlings per panicle were transplanted and grown into M_2_ populations in panicle rows. From the flowering to the mature stage, the plants showing male sterility and/or dwarfism were recorded as putative mutants. For each panicle row with putative mutant plants, 8 WT and 5 putative mutant M_2_ plants were harvested on plant basis. In the following year, M_3_ seeds were grown into plant rows and the respective mutant phenotype was again observed. Once the mutant phenotype was confirmed, M_3_ plants were harvested as done in M_2_ and further grown into M_4_ populations as in M_3_ in the experimental farm of Zhejiang Zhijiang Seed Co. in Yuhang, Hangzhou, Zhejiang Province.

To examine the induced mutation at a molecular level, six M_4_ mutant plants of DS551, two each from Ar, C and Ne ion beam radiation, were selected as representatives for the whole genome sequencing. They were designated as following: Ar_50, Ar_100, C_150, C_200, Ne_50, Ne_100 for the mutated plants according to the type of ion beams and the doses applied, respectively (Table 1). One control rice plant developed from the same progenitor DS551 plant as the mutant plants over the course was also used for sequencing.

### 4.2. Genome Sequencing

The genomic DNA of the WT and six mutant plants were extracted from flag leaf tissues at the flowering stage and fragmented to about 350 bps by a DNA ultrasonic disruptor for the construction of sequencing libraries according to the manufacturer’s instructions (Covaris, Massachusetts, MA, USA). Short paired-end (PE) reads (150 bps) were generated using the Illumina HiSeq 4000 sequencing platform by Novogene Bioinformatics Technology Co., Ltd. (Beijing, China). Raw reads were pre-processed and deposited in the National Center for Biotechnology Information (NCBI) (BioProject ID: PRJNA594450).

### 4.3. Mutation Detection and Annotation

The overall process of mutation detection pipelines is presented in Figure 8. Raw PE reads were filtered using the NGSQCToolkit v 2.3.3 [35], where the cut-off value for PHRED quality score was set to be 20 and for the percentage of read length to be 70. The filtered data were then mapped to the reference Nipponbare genome [36] using the BWA v 0.7.15 [37] with default settings. Subsequently, Sequence Alignment/Map (SAM) files were created for each plant. SAM files were further converted into binary SAM (BAM) files and sorted using the SAMtools v 1.8 [38]. Moreover, PCR duplicates were marked by MarkDuplicates in Picard tools v 1.119 [39], and the coverage and average depth of the reads were calculated using the SAMtools v 1.8 [38], together with an in-house Perl script [40].

The BAM files were further aligned for variation detection using the GenomeAnalysisTK (GATK) v 3.6 [41]. The local realignment of the reads around InDels was performed by using the module InDelRealigner in GATK [41]. The raw SBS and InDel variants were called by inputting the realigned BAM files, respectively, into the module UnifiedGenotyper in GATK [41], SAMtools v 1.8 [38], and the VarScan v 2.4.4 [42] with default parameters. The raw variants were divided into SBSs and InDels using the module SelectVariants in GATK [41]. To exclude possible background mutations, SBSs and InDels common to any two plants were excluded using an in-house Perl script [43].

The variants identified through the above process were further filtered by an in-house Perl script [44,45,46] according to the different bioinformatic tools to remove variants that did not pass “read depth ≥10 && read depth ≤100 && allele frequency ≥0.25”. Then, the SBSs and InDels identified by the above three bioinformatic tools were compared using the AWK commands and only the common variants were ultimately recorded as induced mutations and kept in the GATK VCF format (those only identified by one or two tools were excluded).

The variations identified above were annotated by ANNOVAR [47] and their genomic distribution was visualized by the Circos program [48]. The validation of the variations was performed using an IGV [34,49].

## 5. Conclusions

The present study demonstrated that ^40^Ar^18+^, ^12^C^6+^ and ^20^Ne^10+^ beams could all effectively induce mutations in rice. When proper doses were used, all ion beam radiation are expected to achieve similar high-mutation frequencies. Multiple panicles should be harvested for the development of M_2_ populations with a desired size from the limited number of irradiated seeds. Genome-wide genetic variations of SBSs and InDels were still present in the M_4_ plants, which demonstrated the mutagenic effect of the ion beams on one hand and suggests non-target mutations exist in selected mutants on the other hand.

## Figures and Tables

**Table 1:** Statistics of the Illumina HiSeq whole genome-sequencing data of DS551 and its six M_4_ mutant plants from the three ion beam radiations.

Material ^1^	No. of Mapped Reads	Mapping Rate (%)	Genome Coverage (%)	Sequencing Depth (×)
DS551	135,748,093	98.79	97.30	53.46
Ar_50	153,313,517	95.63	97.37	59.82
Ar_100	169,292,288	98.47	97.41	66.52
C_150	143,348,549	98.29	97.29	55.29
C_200	154,943,939	98.18	97.30	61.15
Ne_50	161,256,389	98.67	97.34	63.27
Ne_100	164,791,769	98.16	97.34	64.84

^1^ Ar, C and Ne stand for argon, carbon and neon ion beams, respectively; the numbers denote the doses (Gy) applied for the mutagenic treatment.

**Table 2:** Number and characteristics of the mutations identified in the six M_4_ plants from the ion beam radiations of rice genotype DS551.

Mutant ^1^	Total	Single Base Substitution (SBS)		Insertion/Deletion (InDel)	
		Subtotal (Ti/Tv) ^2^	Ex. (Syn/Nonsyn /Stop-Gain/Loss) ^3^	Subtotal	Ex. (Frame-/Nonframe -Shift/Stop-Gain/Loss) ^3^
Ar_50	236	205 (141/64)	6 (2/4/0/0)	31	0 (0/0/0/0)
Ar_100	453	404 (297/107)	2 (1/1/0/0)	49	0 (0/0/0/0)
C_150	256	223 (139/84)	5 (1/4/0/0)	33	0 (0/0/0/0)
C_200	269	228 (145/83)	5 (1/4/0/0)	41	1 (1/0/0/0)
Ne_50	307	275 (156/119)	0 (0/0/0/0)	32	0 (0/0/0/0)
Ne_100	238	197 (128/69)	2 (2/0/0/0)	41	1 (1/0/0/0)

^1^ Ar, C and Ne stand for argon, carbon and neon ion beams, respectively; the numbers denote the doses (Gy) applied for the mutagenic treatment; ^2^ Ti/Tv: transition (C:G>T:A and T:A>C:G)/transversion (C:G>A:T, C:G>G:C, T:A>G:C, and T:A>A:T); ^3^ Ex. (Syn/Nonsyn, stop-gain/loss, frame-/nonframe-shift) refers to the number of SBSs or InDels in the exonic regions that were synonymous, nonsynonymous, stop codon gain or loss, frame-shift or nonframe-shift mutations, respectively.

**Table 3:** Characteristics of the ion beams used for the rice seed irradiation.

Ion Beam	Energy (MeV/u)	LET ^1^ (keV/μm)	Doses (Gy)	Dose Rate (Gy/Min.)
^40^Ar^18+^	500	92	50, 100, 150, 200, 300	7.1
^12^C^6+^	290	13	50, 100, 150, 200, 300	7.7
^20^Ne^10+^	400	31	50, 100, 150, 200, 300	5.5

^1^ LET: linear energy transfer.

## Supplementary Materials

The following are available online at https://www.mdpi.com/2223-7747/9/5/551/s1, Figure S1: Collapsed (above) and expanded (below) visualization of the single base substitution at the position of 33,432,582 of Chromosome 2 with C to T mutation (ratio of mutant allele = 1.00) in Ar_100 rice plant, Figure S2: Collapsed (above) and expanded (below) visualization of the single base substitution at the position of 34,459,733 of Chromosome 4 with G to A mutation (ratio of mutant allele = 1.00) in C_150 rice plant, Figure S3: Collapsed (above) and expanded (below) visualization of the single base substitution at the position of 11,795,998 of Chromosome 6 with G to T mutation (ratio of mutant allele = 0.94) in C_150 rice plant, Figure S4: Collapsed (above) and expanded (below) visualization of the single base substitution at the position of 1,950,599 of Chromosome 12 with A to T mutation (ratio of mutant allele = 1.00) in C_150 rice plant, Figure S5: Collapsed (above) and expanded (below) visualization of the single base substitution at the position of 4,402,323 of Chromosome 3 with G to A mutation (ratio of mutant allele = 1.00) in C_200 rice plant, Figure S6: Collapsed (above) and expanded (below) visualization of the single base substitution at the position of 25,928,670 of Chromosome 4 with C to T mutation (ratio of mutant allele = 1.00) in C_200 rice plant, Figure S7: Collapsed (above) and expanded (below) visualization of the single base substitution at the position of 25,928,671 of Chromosome 4 with C to T mutation (ratio of mutant allele = 1.00) in C_200 rice plant, Figure S8: Collapsed (above) and expanded (below) visualization of the deletion at the position of 522,579-522,584 of Chromosome 12 with GGCGCC to G mutation (ratio of mutant allele = 1.00) in C_200 rice plant, Figure S9: Collapsed (above) and expanded (below) visualization of the single base substitution at the position of 15,210,317 of Chromosome 7 with C to A mutation (ratio of mutant allele = 0.57) in Ar_50 rice plant, Figure S10: Collapsed (above) and expanded (below) visualization of the single base substitution at the position of 21,193,688 of Chromosome 8 with C to G mutation (ratio of mutant allele = 0.44) in Ar_50 rice plant, Figure S11: Collapsed (above) and expanded (below) visualization of the single base substitution at the position of 3,176,338 of Chromosome 12 with A to C mutation (ratio of mutant allele = 0.29) in Ar_50 rice plant, Figure S12: Collapsed (above) and expanded (below) visualization of the single base substitution at the position of 3,176,347 of Chromosome 12 with G to A mutation (ratio of mutant allele = 0.31) in Ar_50 rice plant, Figure S13: Collapsed (above) and expanded (below) visualization of the single base substitution at the position of 25,423,413 of Chromosome 1 with C to T mutation (ratio of mutant allele = 0.37) in C_150 rice plant, Figure S14: Collapsed (above) and expanded (below) visualization of the single base substitution at the position of 7,704,982 of Chromosome 5 with T to G mutation (ratio of mutant allele = 0.54) in C_200 rice plant, Figure S15: Collapsed (above) and expanded (below) visualization of the deletion at the position of 22,189,053–22,189,055 of Chromosome 7 with CGA to C mutation (ratio of mutant allele = 0.52) in Ne_100 rice plant.
