# Supplementary material for: Mutagenic Effect of Three Ion Beams on Rice and Identification of Heritable Mutations by Whole Genome Sequencing
Source: Plants (Basel). 2020 Apr 26;9(5):551. doi: 10.3390/plants9050551 (PMC7284785; doi:10.3390/plants9050551)
Supplement: Supplementary file 1 [file plants-09-00551-s001.pdf]

*Detailed instructions about integrative genomics viewer (IGV) are available at http://software.broadinstitute.org/software/igv/AlignmentData.

File Format: The file format for viewing alignments in IGV is the BAM format, a binary form of Sequence Alignment Map (SAM) format. There are three associated tracks in each figure above: Coverage Track to view depth of coverage (top), Alignment Track to view individual aligned reads (middle) and Reference Track to view the reference sequence (bottom).

Coverage Track: By default IGV dynamically calculates and displays the default coverage track for an alignment file. When IGV is zoomed to the alignment read visibility threshold (by default, 30 KB), the coverage track displays the depth of the reads displayed at each locus as a gray bar chart. If a nucleotide differs from the reference sequence in greater than 20% of quality weighted reads, IGV colors the bar in proportion to the read count of each base (A, C, G, T).

Alignment Track: This section gives an overview of the alignment track. When zoomed in to the alignment read visibility threshold, by default 30 KB, IGV shows the reads. When zoomed in sufficiently, IGV displays a line at the center of the display. At higher resolutions, the center line becomes two lines that frame the bases centered in the display, as shown in the figures above, which are the mutation sites we would like to visualize.

- **Color and Transparency for Individual Bases:** By default, read bases that match the reference are displayed in gray. Read bases that do not match are color coded, and insertions and deletions within reads relative to the reference are marked. Insertions are indicated by a purple I (I) and deletions are indicated with a black dash (–). In the figures above, only mismatched bases are color coded and transparency shading of quality is turned off.

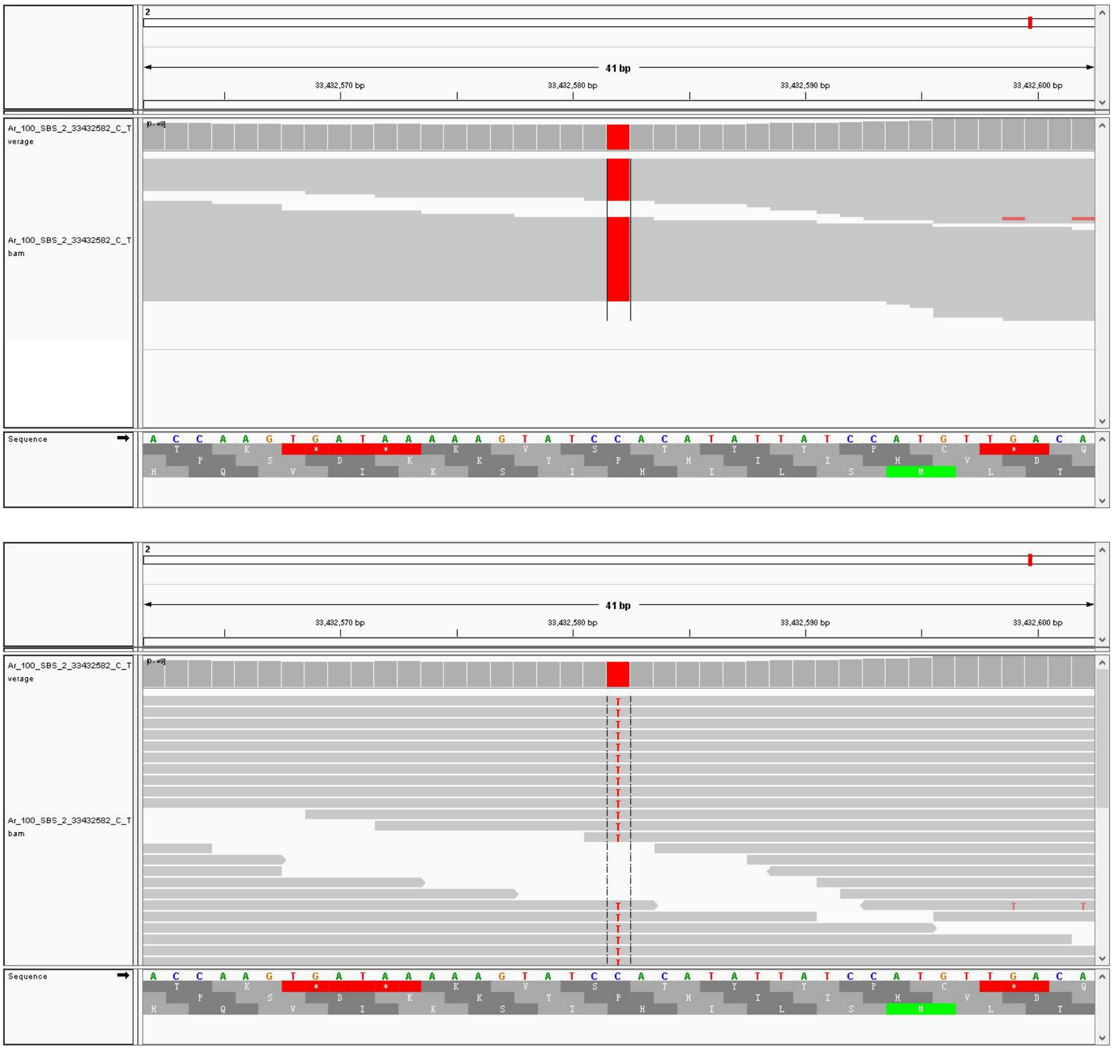

Fig. S1. Collapsed (above) and expanded (below) visualization of the single base substitution at the position of 33,432,582 of Chromosome 2 with C to T mutation (ratio of mutant allele = 1.00) in Ar_100 rice plant.

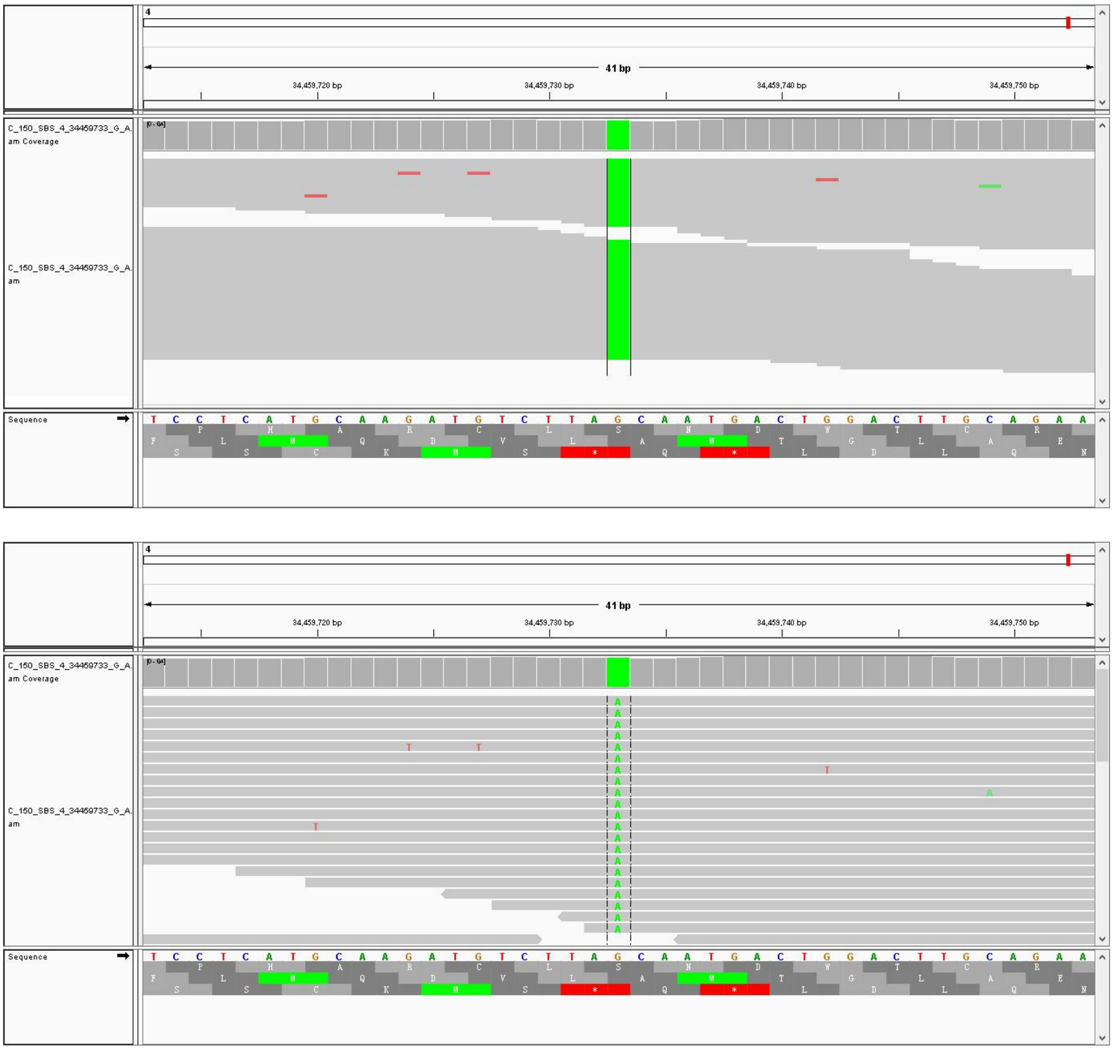

Fig. S2. Collapsed (above) and expanded (below) visualization of the single base substitution at the position of 34,459,733 of Chromosome 4 with G to A mutation (ratio of mutant allele = 1.00) in C_150 rice plant.

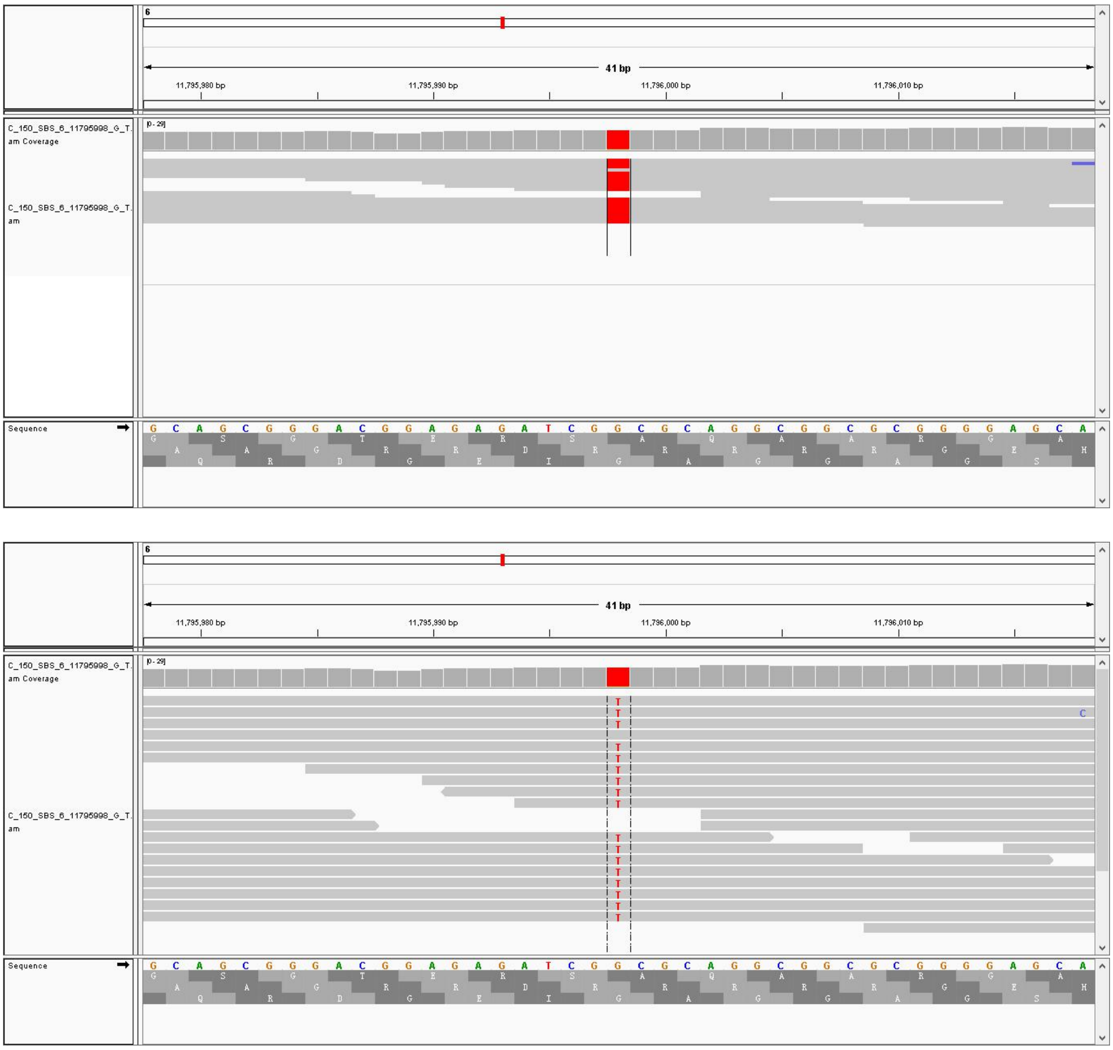

Fig. S3. Collapsed (above) and expanded (below) visualization of the single base substitution at the position of 11,795,998 of Chromosome 6 with G to T mutation (ratio of mutant allele = 0.94) in C_150 rice plant.

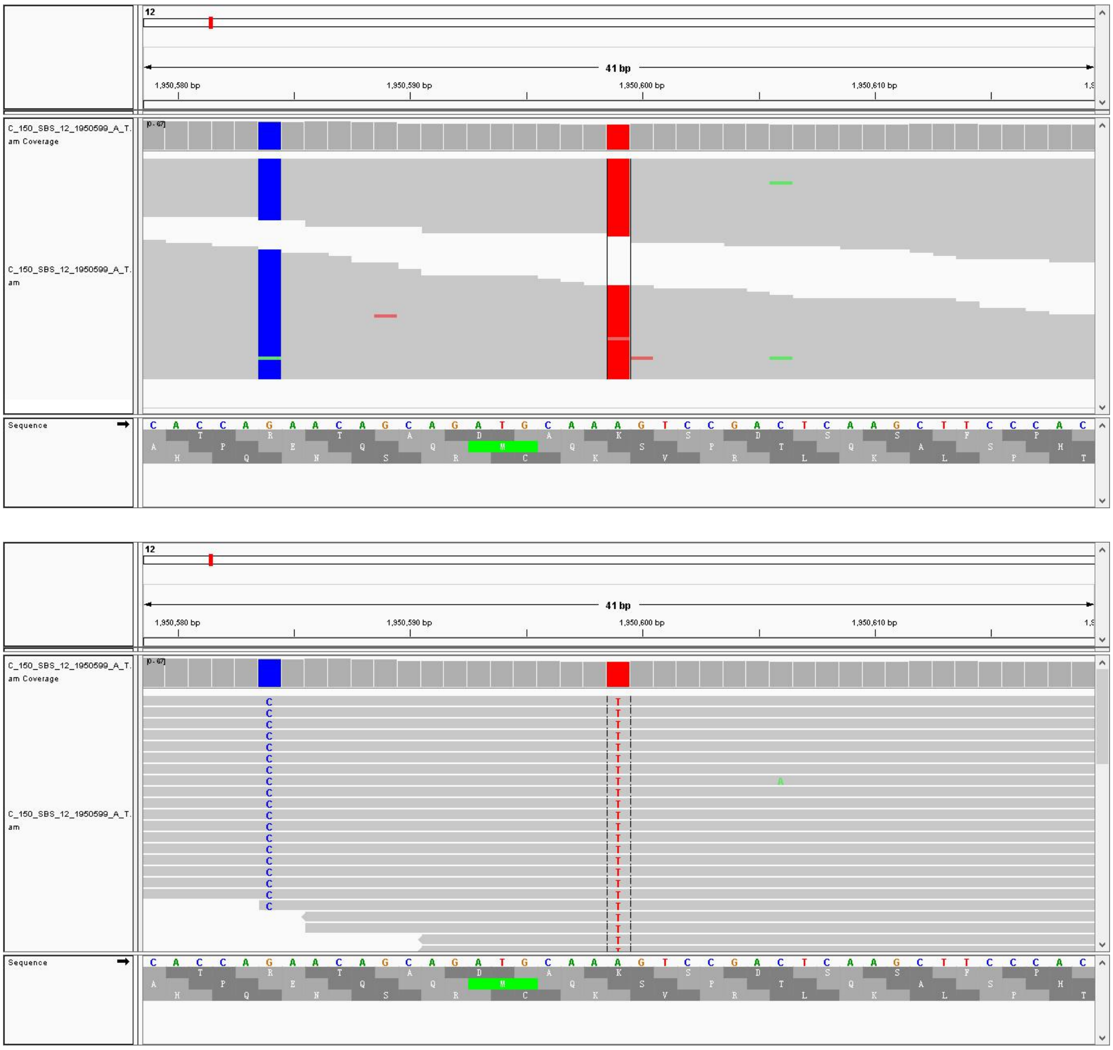

Fig. S4. Collapsed (above) and expanded (below) visualization of the single base substitution at the position of 1,950,599 of Chromosome 12 with A to T mutation (ratio of mutant allele = 1.00) in C_150 rice plant.

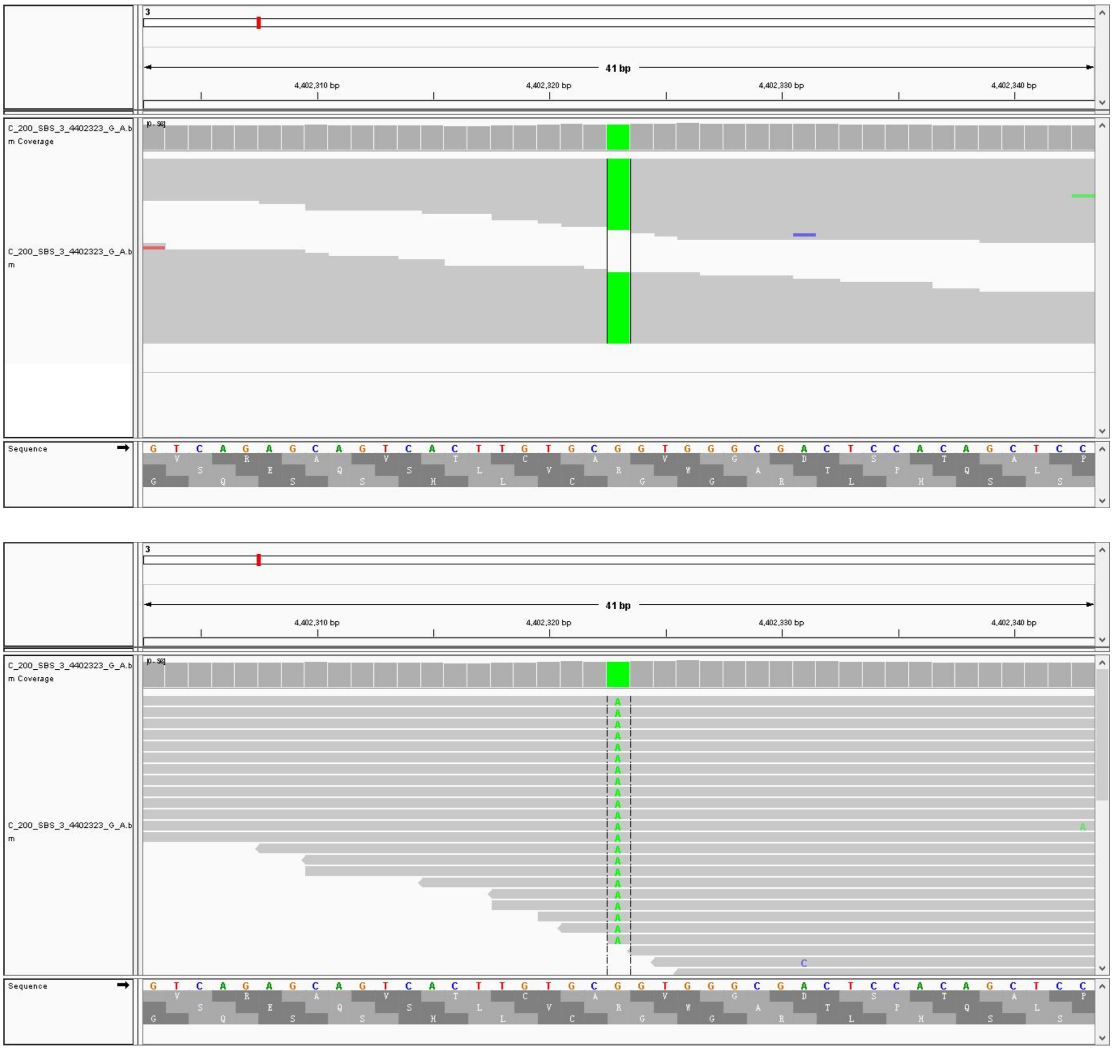

Fig. S5. Collapsed (above) and expanded (below) visualization of the single base substitution at the position of 4,402,323 of Chromosome 3 with G to A mutation (ratio of mutant allele = 1.00) in C_200 rice plant.

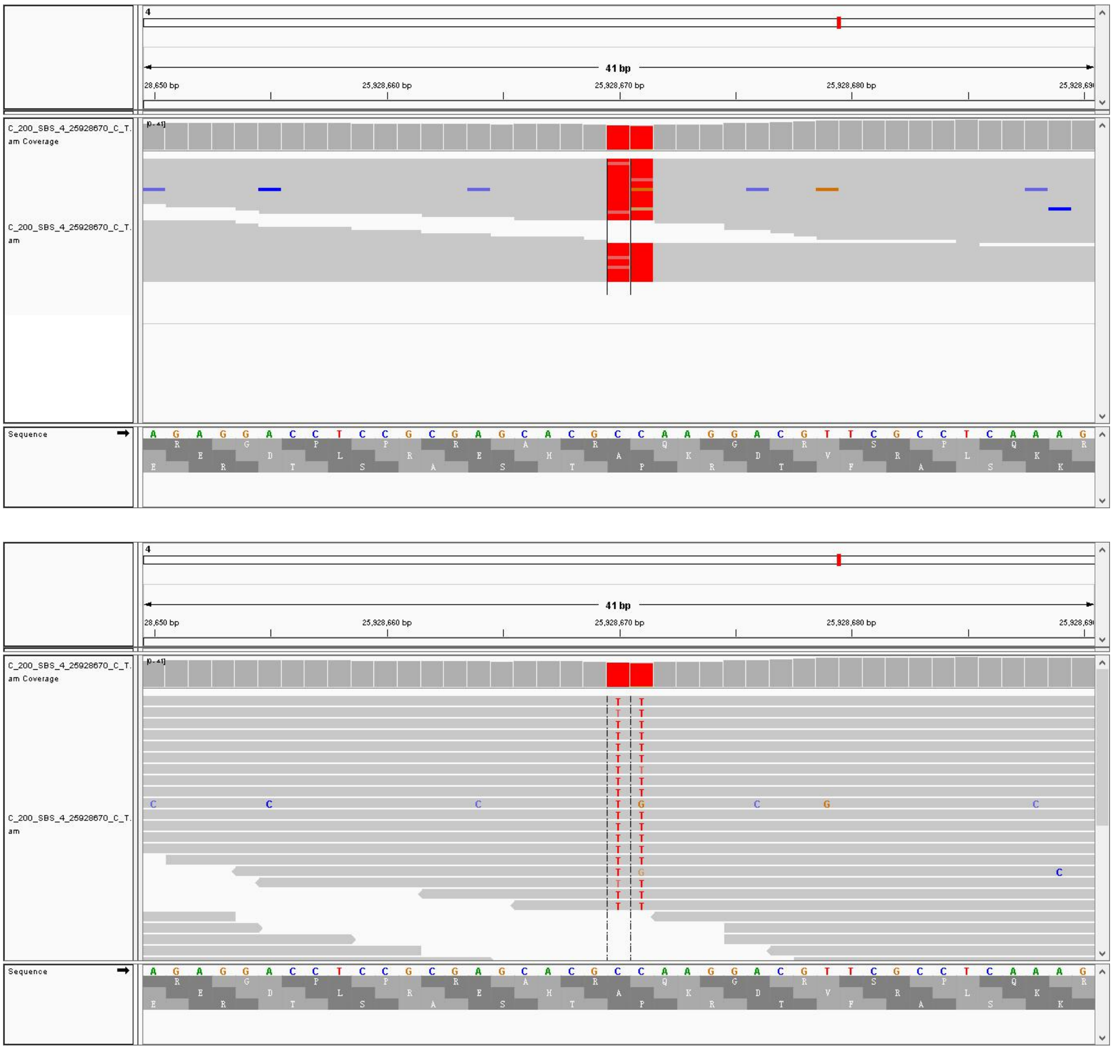

Fig. S6. Collapsed (above) and expanded (below) visualization of the single base substitution at the position of 25,928,670 of Chromosome 4 with C to T mutation (ratio of mutant allele = 1.00) in C_200 rice plant.

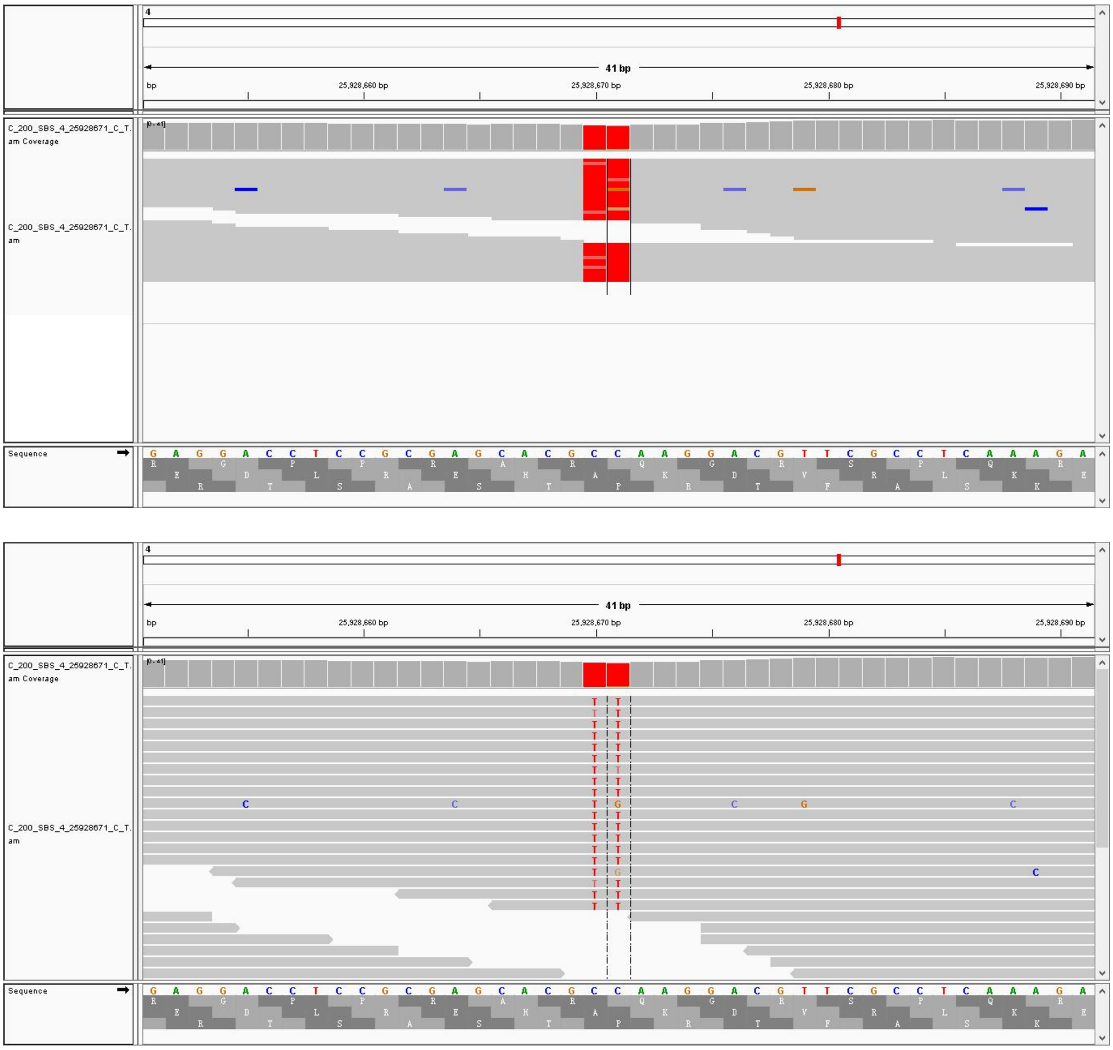

Fig. S7. Collapsed (above) and expanded (below) visualization of the single base substitution at the position of 25,928,671 of Chromosome 4 with C to T mutation (ratio of mutant allele = 1.00) in C_200 rice plant.

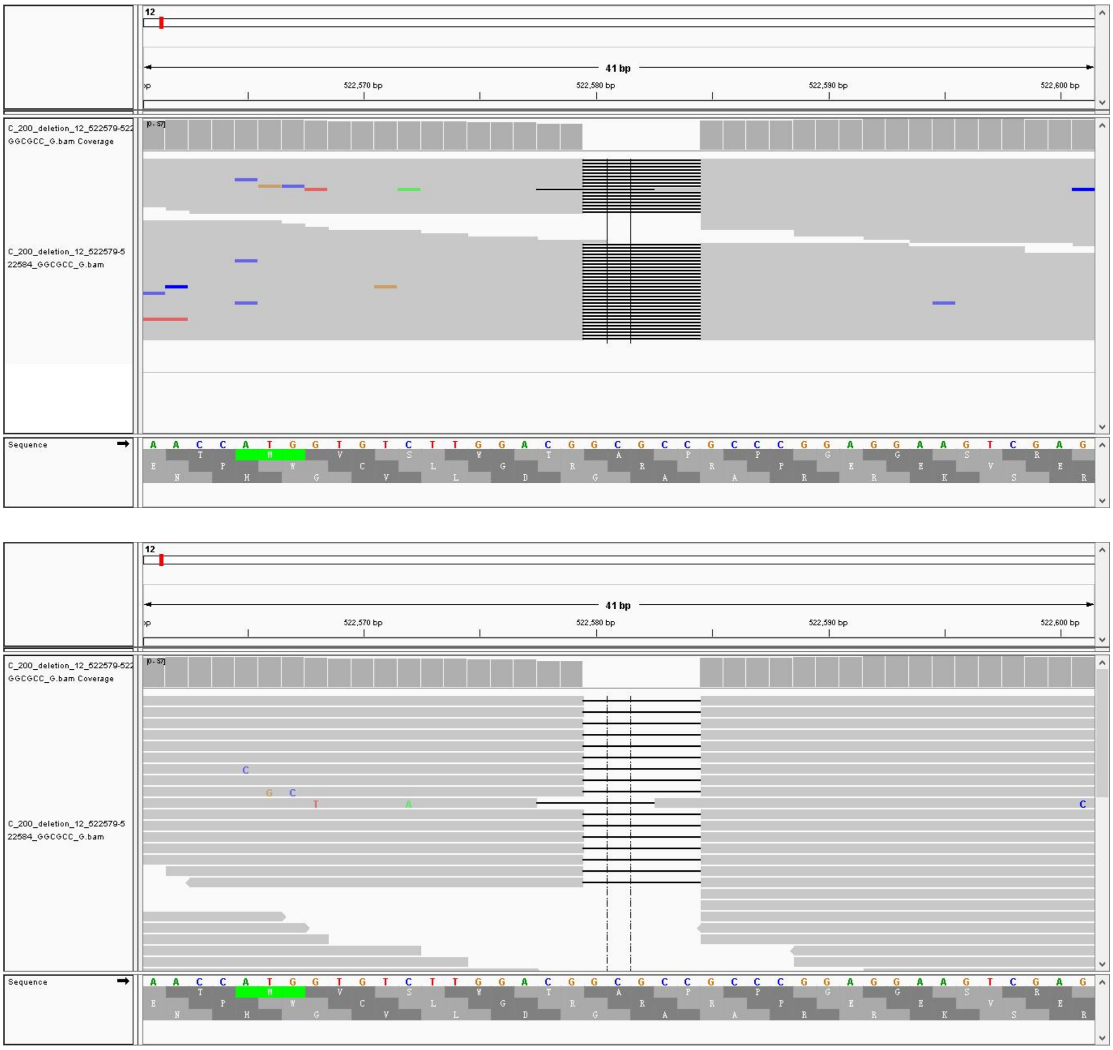

Fig. S8. Collapsed (above) and expanded (below) visualization of the deletion at the position of 522,579-522,584 of Chromosome 12 with GGCGCC to G mutation (ratio of mutant allele = 1.00) in C_200 rice plant.

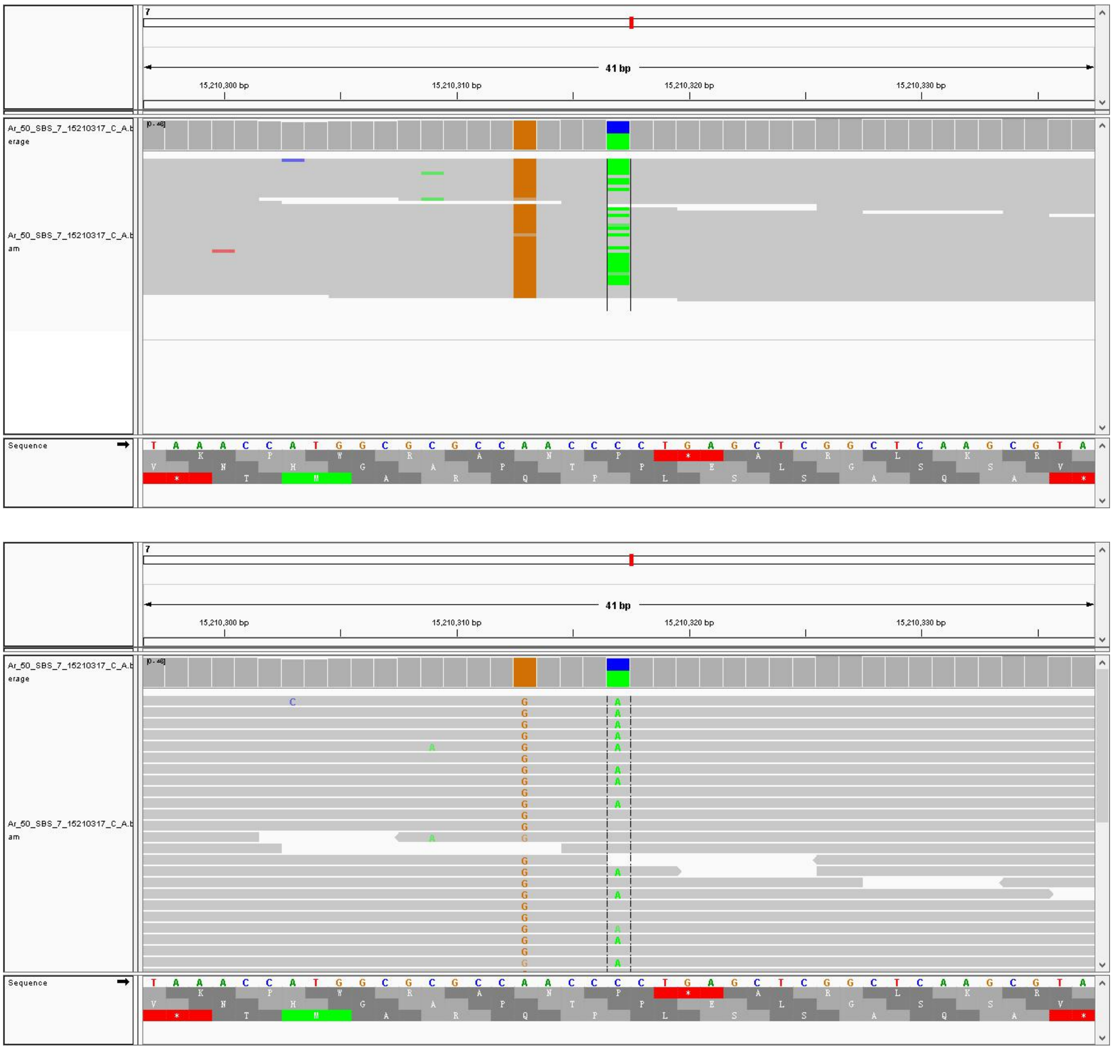

Fig. S9. Collapsed (above) and expanded (below) visualization of the single base substitution at the position of 15,210,317 of Chromosome 7 with C to A mutation (ratio of mutant allele = 0.57) in Ar_50 rice plant.

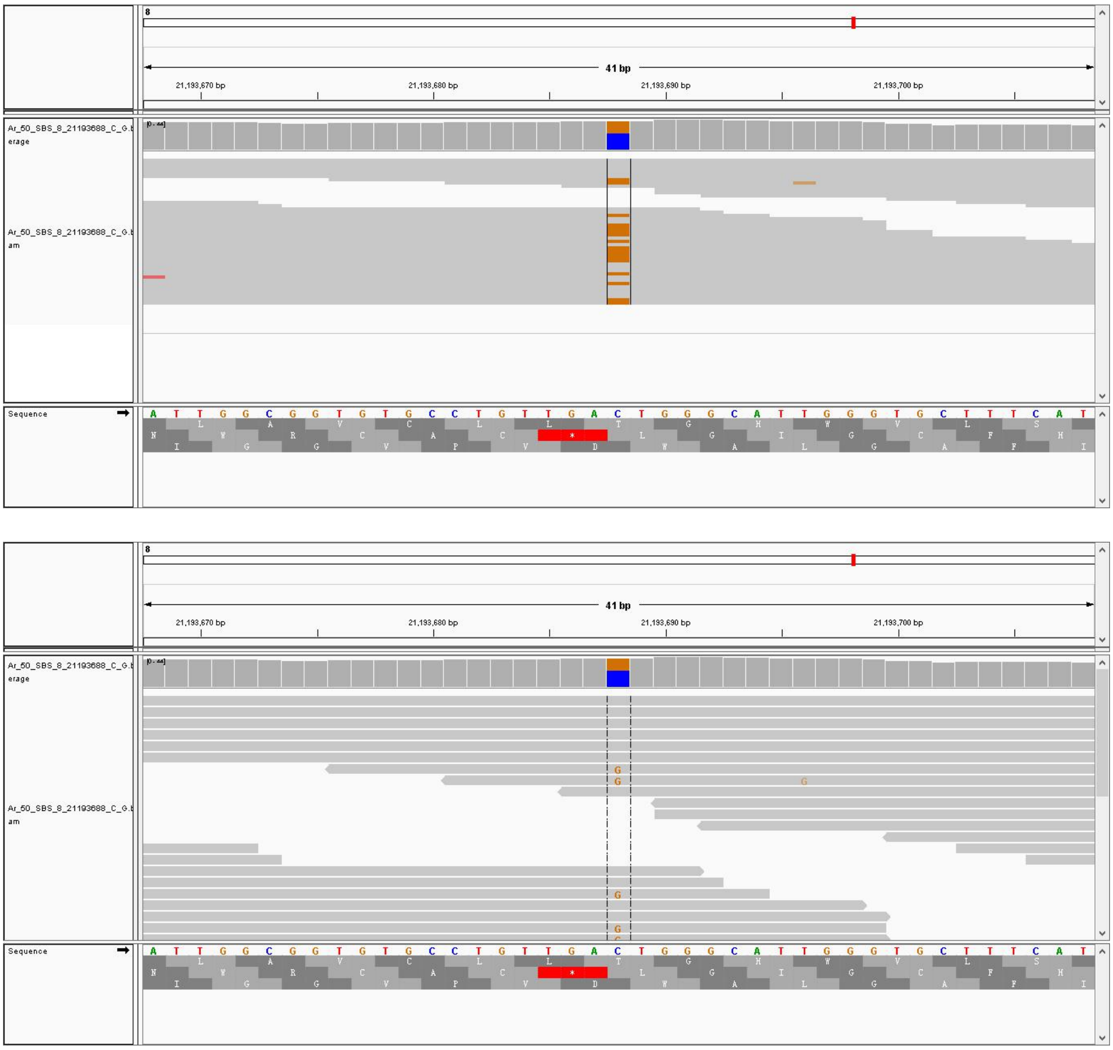

Fig. S10. Collapsed (above) and expanded (below) visualization of the single base substitution at the position of 21,193,688 of Chromosome 8 with C to G mutation (ratio of mutant allele = 0.44) in Ar_50 rice plant.

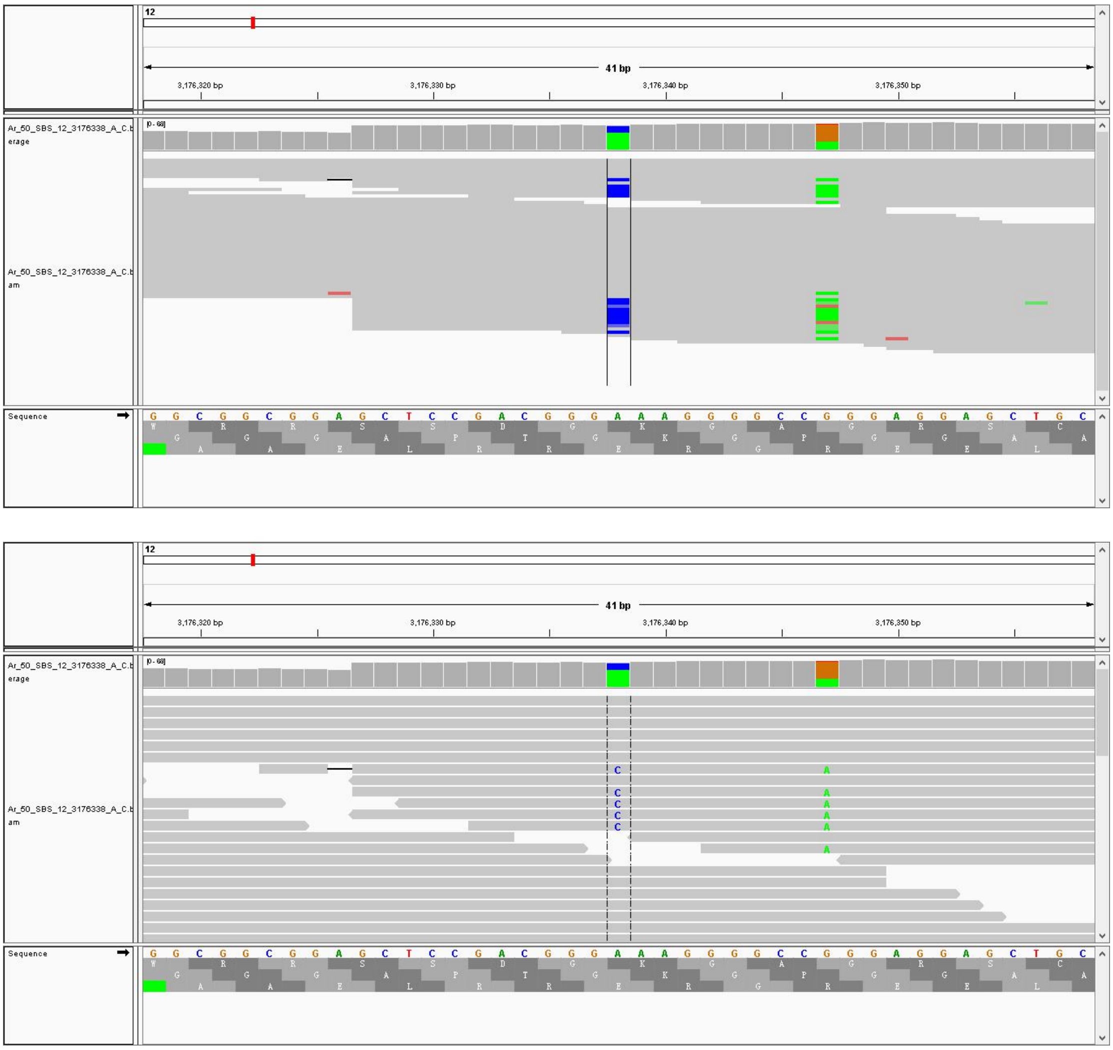

Fig. S11. Collapsed (above) and expanded (below) visualization of the single base substitution at the position of 3,176,338 of Chromosome 12 with A to C mutation (ratio of mutant allele = 0.29) in Ar_50 rice plant.

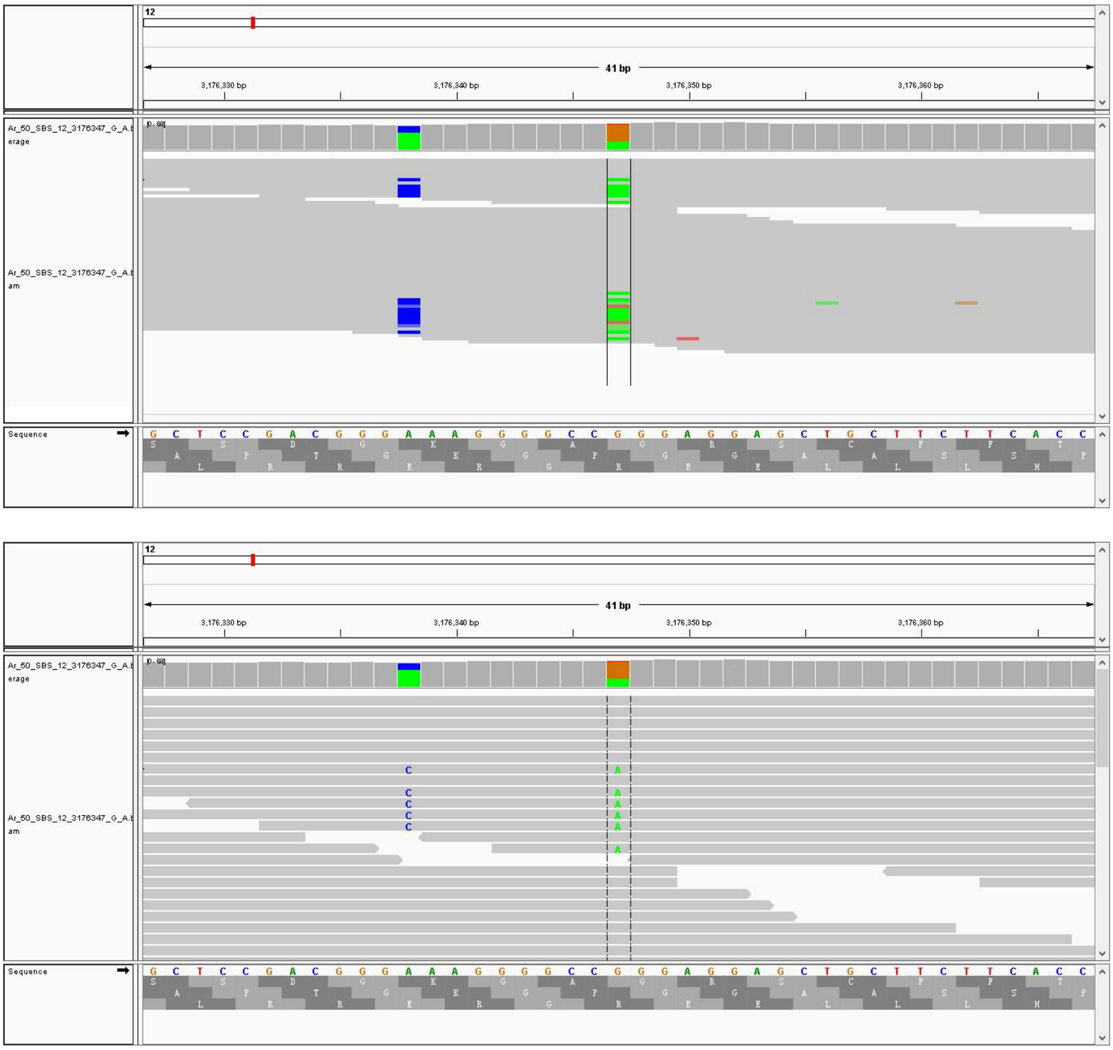

Fig. S12. Collapsed (above) and expanded (below) visualization of the single base substitution at the position of 3,176,347 of Chromosome 12 with G to A mutation (ratio of mutant allele = 0.31) in Ar_50 rice plant.

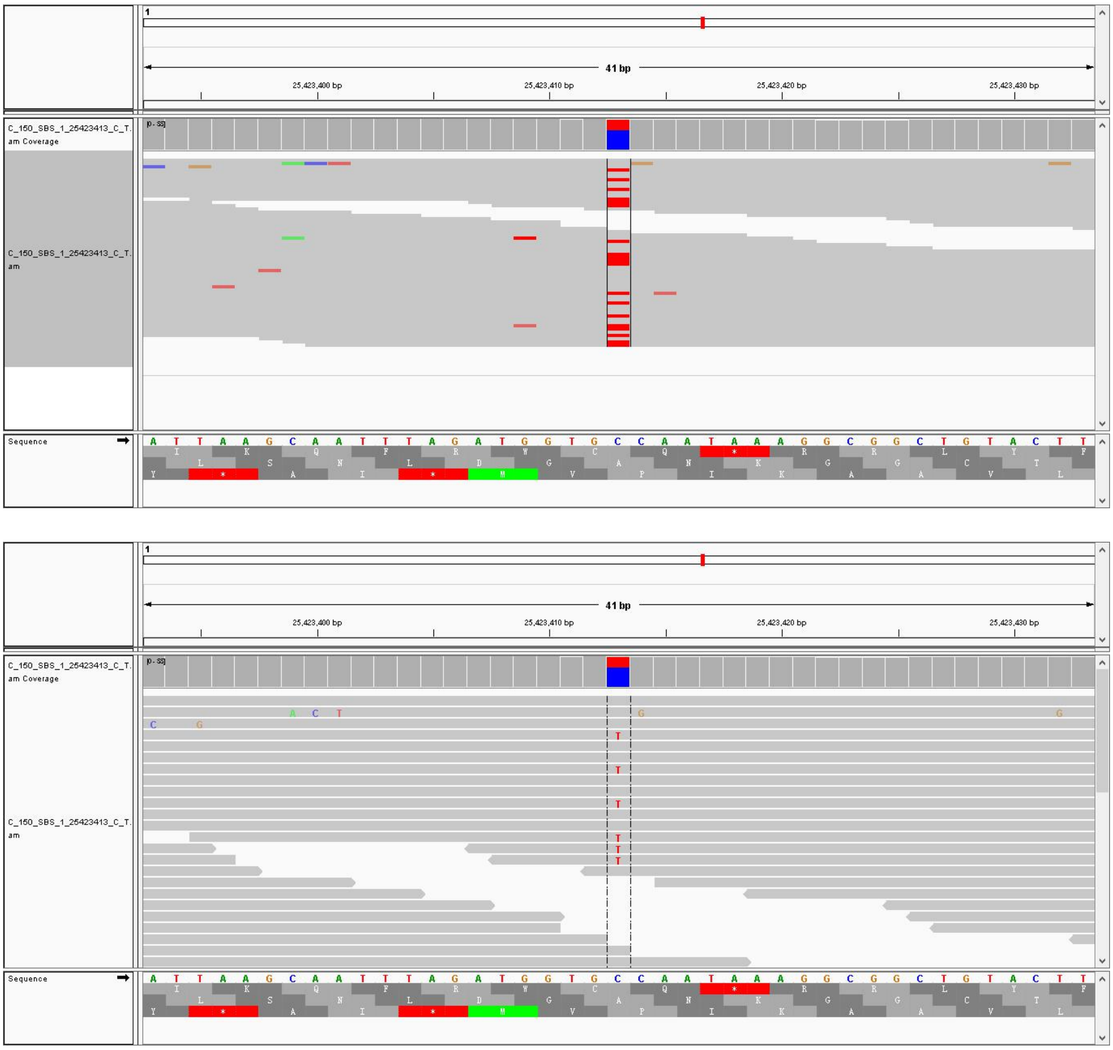

Fig. S13. Collapsed (above) and expanded (below) visualization of the single base substitution at the position of 25,423,413 of Chromosome 1 with C to T mutation (ratio of mutant allele = 0.37) in C_150 rice plant.

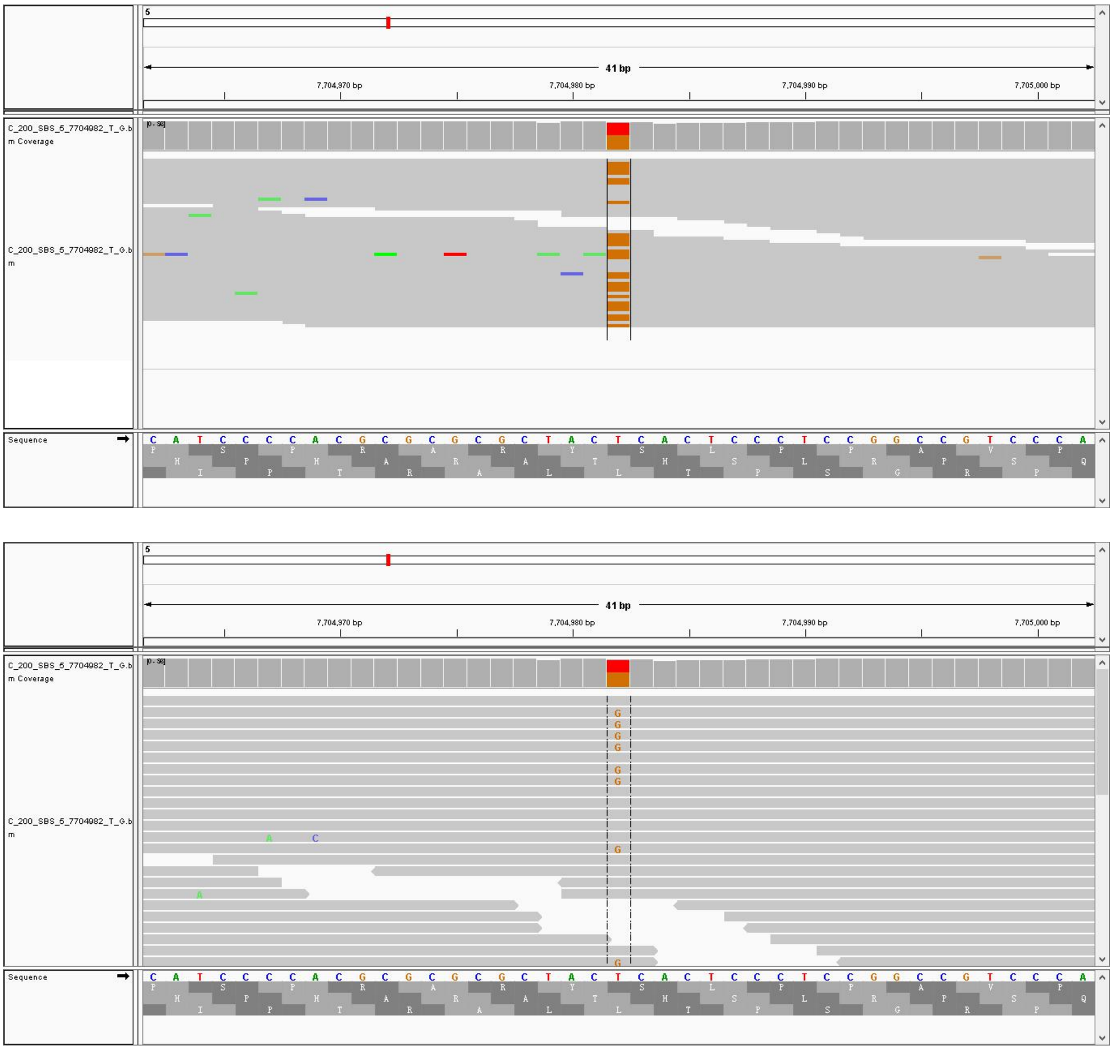

Fig. S14. Collapsed (above) and expanded (below) visualization of the single base substitution at the position of 7,704,982 of Chromosome 5 with T to G mutation (ratio of mutant allele = 0.54) in C_200 rice plant.

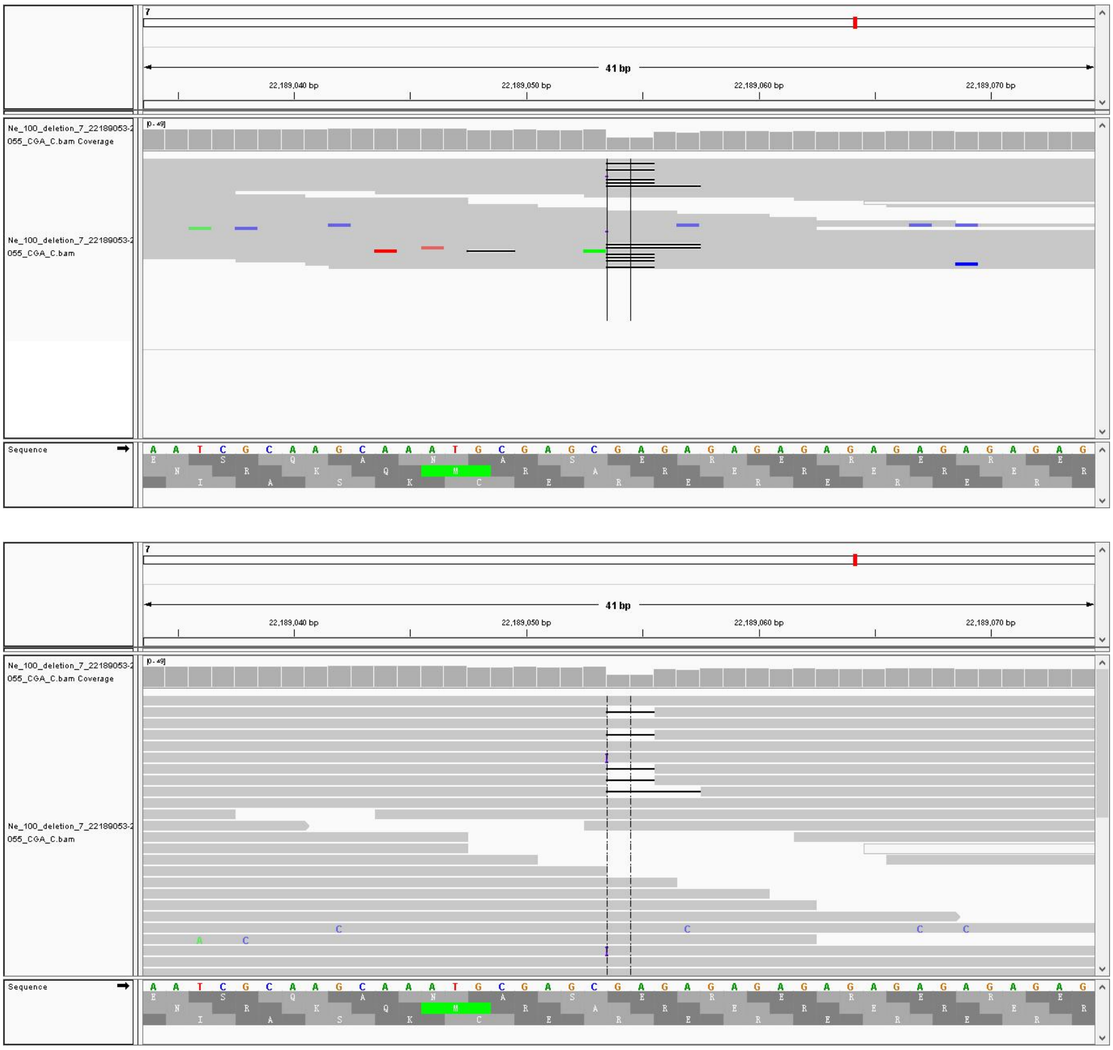

Fig. S15. Collapsed (above) and expanded (below) visualization of the deletion at the position of 22,189,053-22,189,055 of Chromosome 7 with CGA to C mutation (ratio of mutant allele = 0.52) in Ne_100 rice plant.
